# Dengue Incidence Following Mass Vaccination: An Interrupted Time Series Study in Paraná, Brazil

**DOI:** 10.3390/tropicalmed11010011

**Published:** 2025-12-30

**Authors:** Magda Clara Vieira da Costa-Ribeiro, Elias Teixeira Krainski, Angela Maron de Mello, Denise Siqueira de Carvalho, Karin Regina Luhm, Fredi Alexander Diaz-Quijano, Sonia Mara Raboni, Lineu Roberto da Silva, Marilene da Cruz Magalhães Buffon, Eliane Mara Cesário Pereira Maluf, Gabriel Graef, Gustavo Araújo de Almeida, Clara Preto, Silvia Emiko Shimakura

**Affiliations:** 1 Postgraduate Program in Microbiology, Parasitology and Pathology, Department of Basic Pathology, Federal University of Paraná, Curitiba 80060-000, Brazil; 2 Postgraduate Program in Public Health, Federal University of Paraná, Curitiba 80060-240, Brazil; denisecarvalho@ufpr.br (D.S.d.C.); karin.luhm@ufpr.br (K.R.L.); marilenebuffon@ufpr.br (M.d.C.M.B.); almeida.gustavo51@gmail.com (G.A.d.A.); clarampreto@hotmail.com (C.P.); 3 Statistics Program, Computer, Electrical and Mathematical Sciences and Engineering Division, King Abdullah University of Science and Technology, Thuwal 23955-6900, Saudi Arabia; eliaskrainski@gmail.com; 4 Health Ministry, Brasília 70058-900, Brazil; angela.maron@gmail.com; 5 Department of Public Health, Federal University of Paraná, Curitiba 80060-240, Brazil; sraboni@ufpr.br (S.M.R.); lineu.roberto@ufpr.br (L.R.d.S.); eliane.cesario@ufpr.br (E.M.C.P.M.); 6 Laboratory of Causal Inference in Epidemiology—LINCE-USP, Department of Epidemiology, School of Public Health, University of São Paulo, São Paulo 01246-904, Brazil; frediazq@usp.br; 7 Foundation of the Federal University of Paraná (FUNPAR), Curitiba 80010-200, Brazil; gabriel_graeff@hotmail.com; 8 Department of Statistics, Federal University of Paraná, Curitiba 81531-890, Brazil

**Keywords:** dengue, interrupted time-series, vaccine, Dengvaxia^®^

## Abstract

In southern Brazil, dengue transmission in the state of Paraná has shown a significant increase in the number of cases since the first recorded occurrence in 1995, with more frequent outbreaks in the west, northwest, and north of the state. We evaluated the impact of a campaign of dengue vaccination administered to a fraction of the population in 30 municipalities in the state by conducting a 15-year interrupted time-series ecological study using data obtained from an official Brazilian data register. We modeled dengue incidence using Poisson regression adjusted by covariates (demographic, climate, and epidemiological factors), allowing for specific temporal variation for each site. A reduction of 18.7% in dengue incidence rate was estimated for a vaccination coverage of 100%. Although there was an increase in the crude dengue incidence rate, considering the three-dose coverage achieved in the municipalities, we estimated an 8.2% relative reduction in the incidence rate. This reduction would increase to 17% with a hypothetical coverage of 90%. The campaign was more effective in small municipalities since they had higher vaccination coverage. These findings underscore the significant impact of the vaccination campaign on reducing dengue incidence trends across the targeted municipalities.

## 1. Introduction

In Brazil, dengue has spread since 1986, following an epidemic caused by dengue virus serotype 1 (DENV-1) in the state of Rio de Janeiro [1]. This spread is due to the reintroduction [2] and expansion of the main vector, *Aedes* (*Stegomyia) aegypti* (Linnaeus, 1762), as well as factors such as human migration; the occurrence of different serotypes (DENV-1, DENV-2, DENV-3, and DENV-4) and genotypes of the dengue virus [3]; social factors such as population impoverishment; and geographical factors such as climate and environmental changes. Dengue outbreaks follow a cyclical pattern, occurring every three or five years, and the number of cases has been increasing considerably [4]. This indicates that current control measures are not effective enough. The regularity of these cycles, with serotypes alternating between cycles, also suggests the presence of susceptible individuals in the community.

In Paraná state (22°30′58″ to 26°43′00″ S; 48°05′37″ to 54°37′08″ W), which is in southern Brazil, the number of reported dengue cases has been increasing since the first cases were recorded in 1995. Municipalities have reported cases on an annual basis, with the western, northwestern, and northern regions of the state experiencing a higher frequency of dengue circulation. In 2016, all four serotypes of dengue were registered simultaneously. Despite the control measures (cleaning campaigns, insecticide application and educational activities), the state of Paraná has experienced a progressive increase in dengue cases over a period of 21 years [5]. To address this issue, the state opted to vaccinate the population against dengue in 2016 with CYD-TDV (Chimeric Yellow Fever Virus-Dengue Tetravalent Vaccine) Dengvaxia^®^, the first and only licensed vaccine at the time by the Brazilian National Health Surveillance Agency (Agência Nacional de Vigilância Sanitária—ANVISA) [6]. Thirty priority municipalities with the highest incidence (above 300 per 100,000 inhabitants) recorded in the last five years were selected, and specific age groups for vaccination were targeted. Paraná became second in the world, after the Philippines, in administering the dengue vaccine through the public health system. 

Although a previous study has shown that CYD-TDV reduces the risk of dengue among seropositive individuals [7], surveillance data from Paraná revealed a substantial increase in dengue incidence in the years following the mass vaccination campaign. This general rise—likely driven by broader epidemiological dynamics, including the co-circulation of multiple serotypes and the direct and indirect effects of the COVID-19 pandemic—poses challenges to a straightforward assessment of the vaccination impact. Therefore, a key motivation of the present study was the need to estimate the specific contribution of the vaccination campaign on dengue incidence trends by constructing an appropriate counterfactual capable of distinguishing the effect of CYD-TDV from concurrent upward trends in dengue transmission. To address this, we applied a time-series modeling framework incorporating contextual factors and comparisons with unvaccinated municipalities within the same health regions.

## 2. Materials and Methods

This interrupted time series ecological study was conducted using data obtained from Brazil’s Notifiable Diseases Information System (Sistema de Informação de Agravos de Notificação-SINAN) [8,9,10]. SINAN is a computerized system developed by the Brazilian Ministry of Health for registering mandatory notifiable diseases, including dengue fever [11].

A historical record of dengue fever cases was compiled beginning in 1995. However, the time series analysis was restricted to the period from epidemiological week 1/2008 (1 January 2008) to 36/2022 (5 September 2022). This analysis focused on 10 out of the 22 health regions (Regionais de Saúde; RSs) of the Health Department of Paraná State (SESA/PR): the 1st, 9th, 10th, 12th, 14th, 15th, 17th, 18th, 19th, and 20th RSs. These health regions encompass the 30 municipalities where a portion of the population received the dengue vaccine CYD-TDV (Dengvaxia^®^) between 16 August 2016, and 18 December 2018.

**Figure 1:**
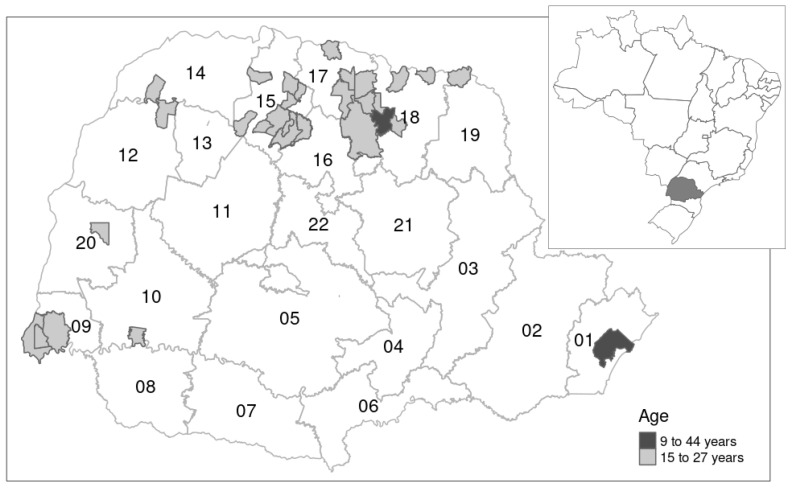
Geographical distribution of vaccinated municipalities in Paraná state, Brazil. Vaccination age range (light gray and black); Health Regions (RS) numbered from 01 to 22 [11].

Of the 30 municipalities selected (with >300 DENV cases per 100,000) for the vaccination program, 5 are in the western region, 24 are in the northern region, and 1 is in the eastern region. The vaccine was administered to the population aged between 9 and 44 years in Assaí (18th RS) and Paranaguá (1st RS), and to those aged between 15 and 27 years in the remaining 28 municipalities. Ages were calculated as of August 2016, the start of the vaccination campaign [11] (Figure 1).

For each of the 10 RSs, the municipalities that did not receive the vaccine were combined and used as control groups to comparatively assess the impact of the vaccine. The analysis included data from 40 locations, consisting of 30 vaccinated municipalities and 10 groups of unvaccinated municipalities within the 10 targeted RSs. The study analyzed data across three age ranges: (a) cases below the vaccination age range, (b) cases within the vaccination age range, and (c) cases above the vaccination age range. The age ranges were defined according to temporal evolution, with the age range increasing by 1 year each year after 2016. The analysis began when the person received their first vaccine dose (Table 1), and the aim was to track the migration of the age ranges over the course of the study period.

### 2.1. The Reference Population for Calculating the Incidence Rate

The population data for each municipality used in this study were obtained from the Brazilian Institute of Geography and Statistics (Instituto Brasileiro de Geografia e Estatística; IBGE) for the years 2008–2009 and 2011–2021, via the IBGE Automatic Recovery System (Sistema IBGE de Recuperação Automática). The IBGE is a federal government agency responsible for collecting annual population data for all municipalities in the country. The population reference for the year 2010 was based on the census conducted that year and divided by age groups. For the year 2022, a growth rate of 9.94% was applied, with the estimated numbers based on the population of previous years as to the date of this analysis in the 2022 Census Reports that had not yet been released. The resulting data include population numbers for each municipality and year, which are divided into three age groups (Supplementary Material S1).

### 2.2. Time Series Model

The output of the adopted model is the weekly count of dengue cases in each of the 40 locations, segmented by age group, resulting in 120 weekly time series. To model the relative incidence risk, we used the population data aggregated by age group and year (Supplementary Material S1).

The statistical model was adjusted for the time series, *y_ikt_*, with *i* = 1, …, 40, indicating the location *i*; *k* = 1, 2, 3 representing the age range and *t* = 1, ..., 766, indicating week *t*, where *t* = 1 is the first epidemiological week of 2008 and *t* = 766 is the 36th epidemiological week of 2022.

The expected number of cases, assuming that the population in location *i*, age group *k*, and epidemiological week *t* has a similar rate to the overall gross rate of the 10 RSs, is calculated as

*E_ikt_* = *m*_0_ × *P_ika_*/*n_a_*, (1)

where *P_ika_* is the population in location *i*, age group *k*, and year *a*, which is repeated for every epidemiological week *t* within year *a*; *n_a_* is the number of epidemiological weeks in year *a*; and *m*_0_ is the annual gross rate (baseline rate) obtained from the absolute incidence in the 10 RSs over the entire period, obtained by

(2) $$m_{0}=\frac{\sum_{i=1}^{40}\sum_{a=1}^{15}\sum_{k=1}^{3}y_{ika}}{\sum_{i=1}^{40}\sum_{a=1}^{15}\sum_{k=1}^{3}P_{ika}},$$

where *y_ika_* is the number of dengue cases in location *i*, age group *k*, and year *a*, for *k* = 1, 2, 3; *a* = 1, ..., 15 (with *a* = 1 being the year 2008) and *i* = 1, ..., 40.

A Poisson distribution was assumed for y_ikt_

*y_ikt_* ∼ Poisson(*E_ikt_* × *λ_ikt_*),

where *λ_ikt_* is the hazard ratio of location *i*, age group *k* relative to the baseline rate at week *t*.

The factors adjusted in the model for the hazard ratio *λ_itk_* were exposure (the weekly coverage of the full course of three doses of vaccination in each municipality); control variables (the circulating serotype in each RS per year); age group (with the vaccinated group as the reference group); a specific temporal variation for each location; and a climate variable calculated from an estimated time series of daily minimum temperature in each location. The climate variable used was the proportion of time in the week when the minimum temperature was above 21 °C, lagged by week (from 0 to 15 weeks) [12,13,14,15,16,17,18,19] (Supplementary Material S2).

### 2.3. Vaccination Coverage

To evaluate whether the vaccination coverage with three doses is associated with the number of cases, an index was calculated that varies over time; the accumulated proportion of the target population vaccinated with three doses (VC), which reflects the weekly vaccination coverage of three doses for each municipality. Coverage is zero before the start of vaccination and reaches the maximum level (three doses) of that municipality at the end of the vaccination period, December 2018.

### 2.4. Vaccination Effectiveness

After adjusting the model, we used Formula (3) to quantify the effectiveness of the vaccine campaign in a municipality with a VC × 100% coverage of three doses of the vaccine:

effectiveness = (1 − exp(β × VC)) × 100%(3)

where β represents the estimated effect of vaccine coverage, and VC (0 ≤ VC ≤ 1) is the coverage rate of three vaccine doses.

### 2.5. Predictions from Two Scenarios

To compare the effectiveness of the vaccine, the model was used to predict the expected number of cases in two hypothetical scenarios for the target population of the vaccine in the 30 municipalities:

Scenario 1: No vaccine dose was applied (0% coverage).

Scenario 2: High percentage of the population in the vaccine age group received three doses (90% coverage).

To predict the number of cases under these scenarios, we used 3000 independent samples (Monte Carlo) from the approximate joint distribution of all model parameters. These scenarios were evaluated from 2 January 2019 (15 days after the end of the vaccination program), to 5 September 2022 (end of the study).

This procedure allows for hypothetical scenarios to be drawn and predicts the expected vaccination effects on the number of dengue cases under varying vaccination coverage conditions, assuming that other conditions, such as circulating serotypes and weather conditions, remain as observed.

In Scenario 1, we determine the reduction in the number of cases attributed to the vaccination campaign by subtracting the observed number of cases from the number of cases predicted by the model under a 0% coverage assumption. This considers each location’s final three-dose coverage rate. To calculate the reduction in cases in Scenario 2, we compare the predicted number of cases under a 90% coverage scenario (which simulates a successful vaccination campaign) to the predicted number of cases under a no vaccination scenario. The predicted number of cases also includes 95% credible intervals to account for the observed coverage and predicted cases under both scenarios.

The R package INLA [18], version 22.04.16, was used considering the central composite design (CCD) strategy for the experimental design to integrate with respect to the three hyperparameters of the model. All the statistical analyses were performed via the R statistical analysis system [20].

## 3. Results

### 3.1. Dengue Incidence and Serotypes

**Figure 2:**
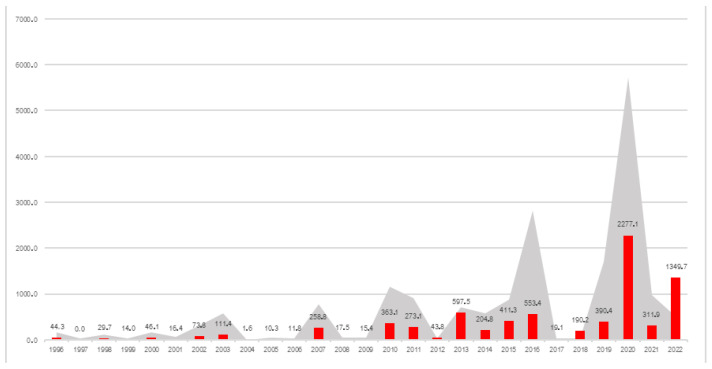
Annual dengue incidence rates per 100,000 inhabitants in 30 municipalities of Paraná state from 1995 to 2022. Municipalities incidence rate (gray); Paraná incidence rate (red).

Paraná state, initially characterized by dengue cases reported in 16 municipalities in 1995, has since expanded to 347 additional municipalities reporting cases as of 2022, resulting in a total of 363 affected municipalities. The vaccinated municipalities were those with consistently high incidence rates, driving the state’s overall pattern (Figure 2).

During the analyzed period, 1,379,141 dengue cases were reported in Paraná, of which 609,632 were of autochthonous origin. After excluding 2161 notifications classified as “discarded” and 482 as “not assessed”, 606,989 probable autochthonous cases were observed in the state, hereafter referred to as “cases” (Table 2).

The 30 municipalities selected for vaccination had 271,114 cases during the specified period. The other 172 municipalities in the same 10 RSs where the vaccination campaign took place that did not participate in the campaign reported 293,940 cases. The remaining municipalities in Paraná had 41,935 cases that were excluded from the analysis, resulting in a total of 565,054 cases (Table 2). Among the municipalities scheduled to receive the vaccine, Londrina had the highest proportion of cases, accounting for 25.1% of the total, followed by Foz do Iguaçu with 23.4% and Maringá with 12.0%, making up a total of 60.5% of the cases. For the unvaccinated municipalities, the cases were more evenly distributed across the 10 RSs, with the highest percentages in the 14th RS (17.8%), the 20th RS (15.4%), the 10th RS (15.2%), the 15th RS (11.7%), and the 12th RS (11.4%) of the cases, accounting for 71.5% of the cases (Table 3).

**Figure 3:**
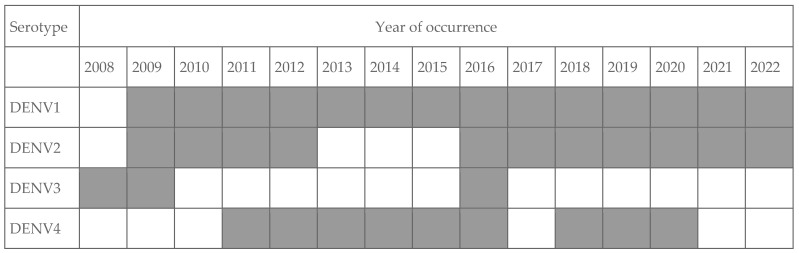
Serotypes identified by year from 2008 to 2022, Paraná state, Brazil. Adapted from [5].

Between 2008 and 2022, the DENV-3 serotype was the least frequently detected (in three years), followed by DENV-4 (in 9 years) and DENV-2 (in 11 years) (Figure 3). DENV-1 was the most prevalent virus in this period and presented high incidence rates, except in 2020, when more than 80% of the identified virus was DENV-2 [5].

**Figure 4:**
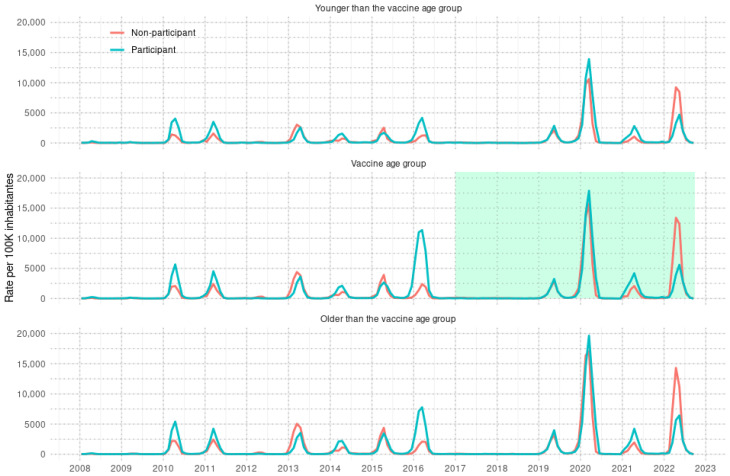
Monthly dengue incidence rates (per 100,000 inhabitants) by age groups in municipalities in Paraná state, participating in vaccination versus municipalities not participating in vaccination from 2008 to 2022. The green area in the vaccine age group panel shows the period of time six months after the beginning of the vaccination.

The group of municipalities selected for vaccination had the highest incidence rate of dengue in 2016 among the age groups eligible for vaccination. From 2019 to 2021, the rates were even higher for all age groups in the municipalities participating in vaccination (Participant) than in the unvaccinated group (Non-participant). However, in 2022, the situation was reversed, with higher dengue rates in the unvaccinated group across all age groups (Figure 4).

The aggregated incidence rates for the pre-vaccine (2008–2015) and post-vaccine (2019–2022) periods by age group (vaccine age range, below and above the vaccine age range), as well as the RRs, are presented in Table 4. The incidence rates during the post-vaccine period were higher than those during the pre-vaccine period, both within and outside the vaccine age range. However, the magnitude of the RR was lower in municipalities where vaccination was conducted in each age group. For example, in the targeted age range, the RR for the post-vaccine period compared with the pre-vaccine period was 2.4 in vaccinated municipalities, whereas it was 4.4 in unvaccinated municipalities.

Weekly time series of incidence rates for the three age ranges were higher in the municipalities of Tapira (12th RS), São Jorge do Ivaí (15th RS), Porecatu (17th RS), São Sebastião da Amoreira (18th RS), Cambará (19th RS), and Maripá (20th RS) in 2022 than in unvaccinated municipalities in the same RS. In 2020, in Foz do Iguaçu (9th RS), dengue caused by serotype DENV-2 affected all age groups, including vaccinated individuals, with incidence rates much higher than 300 per 100,000 inhabitants. High rates also occurred in the other two municipalities that received the vaccine; however, in the vaccinated group, they were slightly lower than in the rest of the region. However, in 2022, these municipalities showed higher incidence rates compared to Foz do Iguaçu, where rates were lower, and the predominant virus was DENV-1.

### 3.2. Vaccine Coverage

**Figure 5:**
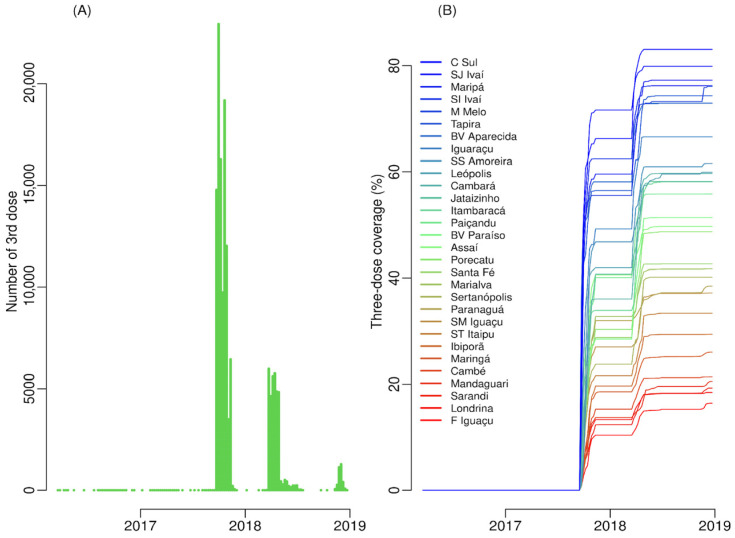
Evolution of the proportion of people vaccinated against dengue, with three doses over time, in 30 municipalities of Paraná state from 2016 to 2018, Brazil. (**A**) Weekly number of people vaccinated with three doses; (**B**) Weekly coverage of three vaccine doses.

The weekly time series of the number of third doses of the dengue vaccine shows three decreasing peaks over time (Figure 5A). The largest peak occurred in the second half of 2017, the intermediate peak at the beginning of 2018, and the smallest at the end of 2018, which marked the finish of the vaccination campaign. The time series of the weekly vaccination coverage for three doses per participating municipality in the vaccination campaign shows significant variability in vaccination coverage (Figure 5B).

### 3.3. Time Series Analysis

The climate variable was adjusted in the model by considering different time lags, and the lags between 9 and 12 weeks showed the best fit. They were combined into a common factor (the proportion of time during the period of 9–12 weeks before the date of the cases when the minimum hourly temperature exceeded 21 °C) and used in the final model [12,13,14,15,16,17,18,19] (Supplementary Material S2).

The estimated effect of vaccination coverage −0.207 (95% CI = [−0.252; −0.161]) was significantly less than zero, resulting in a reduction in the hazard ratio of dengue cases (HR = 0.813; 95% CI = [0.777; 0.851]).

The effectiveness of the vaccine campaign for a (vaccination coverage of 100%) was estimated from this result as 18.7% (95%CI = [14.9%; 22.3%]) [21,22,23] (Supplementary Material S3). This percentage reduction in cases is expected in a hypothetical scenario where a municipality has 100% coverage of the third vaccine dose. However, none of the study locations achieved vaccination coverage of 100%. The municipality with the highest vaccination coverage of three doses was Cruzeiro do Sul, with 83.0%, and in this case, the campaign’s effectiveness was estimated to be 15.8%. In contrast, the municipality with the lowest vaccination coverage was Foz do Iguaçu, with only 16.4% of its population vaccinated, resulting in an estimated effectiveness of $\left(1-exp\left(-0.207\times 0.164\right)\right)\cdot 100\%=3.3\%$.

**Figure 6:**
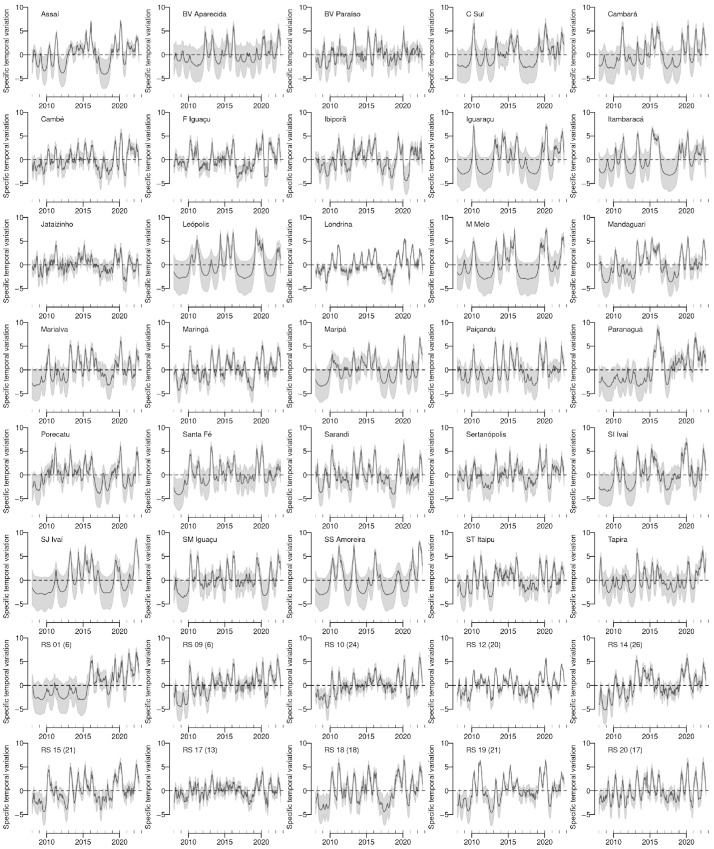
Estimated specific temporal variation for each location (black line) and 95% credible intervals (grey color) in Paraná state, Brazil.

The estimated specific temporal variation for each location after adjusting for all the observed factors, along with their 95% credible intervals estimated under the model, are represented in Figure 6. Different patterns are observed in the series, both in terms of their profiles and error margins. However, neighboring municipalities, such as Maringá and Sarandi and Londrina and Cambé, tended to exhibit similar patterns of cyclical variation in dengue. Interestingly, Paranaguá’s specific temporal variation was low until 2016 and the highest in 2016 compared with the other locations. In fact, dengue was introduced in Paranaguá in 2016, and it had the highest observed rate.

The findings from the first scenario suggest that there was an 8.2% decrease in the number of dengue cases due to the vaccination campaign. This means that there were 3411 fewer cases than what the model had predicted in the absence of the vaccination. For a three-dose vaccination coverage reaching 90% (Scenario 2), the expected overall reduction in dengue cases across all 30 municipalities was 17.0%, corresponding to 7090 fewer cases (Supplementary Material S4).

## 4. Discussion

Between 2008 and 2015, the western, northwestern, and northern regions of the state of Paraná recorded the highest number of dengue cases. It was only in 2016, in the eastern region, specifically in the city of Paranaguá, that the first significant epidemic of DENV-1 occurred on the state coast [24]. The history of DENV infection varied among the 30 municipalities selected for vaccination. Londrina had the highest number of cases (68,036), followed by Foz do Iguaçu (63,440) and Maringá (32,624), whereas Munhoz de Melo (550), Boa Vista da Aparecida (343), and Leópolis (295) had the lowest number of cases. However, the highest incidence until 2016 was observed in small municipalities.

In a previous vaccine effectiveness study using individual-level data, we demonstrated that CYD-TDV (Dengvaxia^®^) was associated with a 21.3% reduction in the risk of dengue, with the effect being modified by a history of dengue [7]. In this new study, we documented the significant impact of the vaccination campaign on the incidence rate trend. As expected, the overall population impact of the campaign was statistically significant, dependent on vaccine coverage, and independent of contextual factors. The proposed model included covariates, such as the circulating serotypes, population density, temperature, and age within the recommended vaccination range.

Our findings indicate that larger municipalities act as modulators of seasonal DENV transmission patterns at the regional level, whereas smaller municipalities (<10,000 inhabitants) show seasonal patterns that are largely determined by the epidemiological context of their surrounding regions.

Temperature is one of the key factors affecting survival, longevity, vector capacity, and other aspects of the life cycle of *Ae. aegypti* [25]. In this study, temperature was identified as a risk factor (HR = 3.49 95% CI = [2.73; 4.49]) for the dengue virus. In a temporal and spatial analysis, the high incidence rates of dengue and the heterogeneous spatial distribution in the state of Paraná were attributed to sociodemographic factors and environmental determinants [26].

For the model, the DENV-1 serotype was the only one that presented a significant risk factor (HR = 3.49 95% CI = [2.73; 4.49]) for the occurrence of dengue epidemics. Proportionally, DENV-1 was the serotype that was present in the state for the longest period. One probable explanation for this is the introduction of new variants and the genetic diversity of DENV-1 [27]. The V genotype (American/African), which is grouped in the clade of lineages I, II, and III, was detected for this serotype in Brazil [28]. The lowest risk was attributed to DENV-3, followed by DENV-2 and DENV-4. DENV-2 was the first serotype to circulate in the southern region of the country, and it was introduced in the state of Paraná in 1995. DENV-1 was introduced in Santa Catarina in 1999, DENV-3 in Paraná in 2000, and DENV-4 in Rio Grande do Sul in 2011 [29].

The highest degree of vaccination coverage was observed in municipalities with fewer than 10,000 inhabitants, as previously reported [11]. Among these municipalities, Cruzeiro do Sul had the highest coverage rate at 83.0%, resulting in a higher estimated effectiveness rate of 15.8% (95% CI = [12.5%; 18.9%]). Conversely, Foz do Iguaçu had the lowest coverage rate at 16.4%, resulting in a lower effectiveness rate of 3.3% (95% CI = [2.6%; 4.0%]). The overall effectiveness rate across all 30 municipalities was 18.7% (95% CI = [14.9%; 22.3%]). Despite the high variability in vaccination coverage, with 33% of municipalities having a coverage rate of 40% or less and low vaccine effectiveness, the two scenario predictions suggest an overall reduction of 8.2% in dengue cases when three vaccine doses are considered. The approach used includes a vaccination coverage indicator that varies over time, ranging from 0% coverage before the start of vaccination to the achieved coverage level in each municipality, which could reach 95% of the target population.

In the analysis of the incidence rates (unadjusted), there was a generalized increase in dengue cases after the vaccination period. However, in the municipalities where the vaccine was implemented, the rate ratio in the vaccinated age group was the lowest (RR = 2.4), suggesting a possible vaccine effect. According to the statistical model, individuals below and above the vaccinated age group presented a lower risk for dengue infections. However, Paranaguá, one of the municipalities that targeted the age group of 9–45 years, experienced the first dengue epidemic between 2014 and 2016 [24]. The co-circulation of multiple dengue serotypes reduces adults’ susceptibility due to acquired immunity from the disease [30]. In Paraná, the highest incidence rate was reported in the population aged between 20 and 59 years, with a significant difference in the incidence rate between age groups: ≤10 years (*p* < 0.01), ≥60 years (*p* < 0.05), and 11–19 years (*p* < 0.05) [26]. Furthermore, a global analysis revealed that individuals under 5 years of age, the age group previously responsible for most deaths and years of life lost due to dengue in 1990, were replaced by those between 15 and 49 years of age in 2019 [31].

Dengue epidemics are complex and multifactorial [26,32]. Brazil is experiencing a hyperendemic scenario, with the co-circulation of all four serotypes of DENV and an increasing occurrence of severe and fatal cases, as well as the co-circulation of other arboviruses, such as Zika, yellow fever, and Chikungunya [31].

The susceptibility of the human population to circulating serotypes and their genotypes, as well as the mosquito infestation index, are factors related to the number of dengue cases [33]. Moreover, climatic, environmental, social, and demographic factors, as well as vector control measures, also play a significant role in its incidence.

Several limiting factors made it difficult to adjust the model during the study. For example, it was challenging to obtain a representative sample of the circulating serotype and its genotypes. In our previous study using individual data from confirmed cases, we reported that the vaccine effectively reduced the risk for certain serotypes (DENV-1 and DENV-4) but had no such effect on DENV-2. In fact, in patients without prior dengue infection, the vaccine increased the risk of DENV-2. Thus, the circulating serotype is a key factor in determining the impact of a vaccine. In the present study, which focused on probable cases (most of which lacked serotype data), we were unable to disaggregate cases by serotype. Nevertheless, the significant reduction in probable cases indicates that the benefits of vaccination outweigh any adverse effects. The heterogeneity of the efficacy of this vaccine has already been documented in clinical trials [34]. Thus, it is well accepted that a seronegative status implies a lack of benefit. Therefore, the vaccine would be indicated only for seropositive individuals. Since the campaign was carried out before this knowledge was available, there would be concern about the population impact of the mass vaccination program. In this sense, our study contributes to assessing the impact from the perspective of its association with the incidence rate of probable cases. These results complement previously published estimates that focused on confirmed cases [7,35].

Additionally, historical data on dengue in the target municipalities and data on the infestation of *Ae. aegypti* were unavailable. Another issue was the limited number of meteorological stations in the state.

It is important to acknowledge that dengue incidence increased markedly across the state during the post-vaccination period, reflecting the influence of factors that were unrelated to the intervention. The COVID-19 pandemic, for example, may have affected viral circulation patterns, health-seeking behavior, reporting practices, and vector control activities, potentially amplifying dengue transmission. Moreover, the predominance of particular serotypes, especially DENV-2 during certain years, may have attenuated the population-level impact of the vaccine, given known differences in vaccine performance by serostatus and serotype.

Despite these strong external pressures, our modeling results indicate that the increase in dengue incidence was smaller than expected in municipalities that implemented vaccination when compared with the estimated counterfactual scenario. In other words, although the crude trends show an overall rise, the vaccination campaign appears to have mitigated part of this increase. This finding aligns with previous evidence of CYD-TDV’s individual-level effectiveness and reinforces that the campaign’s impact should be interpreted as a relative reduction in expected incidence, rather than as prevention of outbreaks under the highly unfavorable epidemiological conditions of recent years.

These observations underscore the importance of accounting for secular trends, serotype dynamics, and concurrent public health disruptions when evaluating population-level vaccine impact. Relying solely on crude pre-post comparisons would risk underestimating the benefits of the campaign, whereas the counterfactual modeling framework allows a more accurate estimation of its marginal effect.

## 5. Conclusions

An analysis of the unadjusted incidence rates revealed a general increase in dengue occurrence during the post-vaccination period across the state. In this context of rising transmission, the time-series model indicated that municipalities implementing the vaccination campaign experienced a lower-than-expected increase in dengue incidence when compared with the counterfactual scenario. After adjusting for serotype circulation, climatic conditions, age structure, and location-specific temporal patterns, vaccination coverage was associated with a significant reduction in the risk of dengue. According to model-based predictions, the campaign prevented an estimated 8.2% of cases under the coverage levels achieved, and this reduction could have reached 17% with higher vaccination coverage. These findings suggest that, despite the strong upward secular trends in recent years, the mass vaccination campaign contributed to mitigating the expected burden of dengue in the targeted municipalities.

## Figures and Tables

**Table 1:** Age groups (years) by year, considering age migration, in the 30 municipalities selected for vaccine administration in Paraná state, Brazil.

All Other 28 Municipalities	Assaí and Paranaguá	Year
15 to 27	9 to 44	2016
16 to 28	10 to 45	2017
17 to 29	11 to 46	2018
18 to 30	12 to 47	2019
19 to 31	13 to 48	2020
20 to 32	14 to 49	2021
21 to 33	15 to 50	2022

**Table 2:** Dengue notifications according to SINAN, Paraná state, from 1 January 2008 to 5 September 2022.

Final Classification	Autochthonous Origin				Total	
	Indefinite	N.A.	No	Yes	*N*	%
Dengue	2008	33,666	17,874	595,212	648,760	47.0
Dengue with warning signs	35	408	199	7458	8100	0.6
Severe dengue	12	40	48	1396	1496	0.1
Inconclusive	395	60,744	492	2923	64,554	4.7
Probable cases	2450	94,858	18,613	606,989	722,910	52.4
Discarded	54	640,163	7113	2161	649,491	47.1
N.A.	6	6211	41	482	6740	0.5
Total	2510	741,232	25,767	609,632	1,379,141	100.0

N.A.: Not Assessed.

**Table 3:** Frequency of dengue cases in municipalities located in the 10 health regions of Paraná state, Brazil, participants and non-participants of the dengue vaccination campaign from 1 January 2008 to 5 September 2022.

Vaccinated Municipalities	*N*	%	Health Region (Unvaccinated Municipalities)	*N*	%
Paranaguá	18,577	6.9	RS 01 (6)	4326	1.5
Foz do Iguaçu	63,440	23.4	RS 09 (6)	16,701	5.7
SM Iguaçu	2964	1.1			
ST Itaipu	4321	1.6			
Boa Vista da Aparecida	343	0.1	RS 10 (24)	44,603	15.2
Tapira	1205	0.4	RS 12 (20)	33,417	11.4
Cruzeiro do Sul	1009	0.4	RS 14 (26)	52,466	17.8
Santa Isabel do Ivaí	3386	1.2			
Iguaraçu	613	0.2	RS 15 (21)	34,361	11.7
Munhoz Melo	550	0.2			
Mandaguari	2520	0.9			
Marialva	3171	1.2			
Maringá	32,624	12.0			
Paiçandu	4086	1.5			
Santa Fé	2299	0.8			
Sarandi	15,830	5.8			
SJ Ivaí	1111	0.4			
Assaí	4205	1.6	RS 17 (13)	28,034	9.5
Bela Vista do Paraíso	2712	1.0			
Cambé	12,575	4.6			
Ibiporã	6500	2.4			
Jataizinho	5448	2.0			
Londrina	68,036	25.1			
Porecatu	4020	1.5			
Sertanópolis	4346	1.6			
Itambaracá	649	0.2	RS 18 (18)	21,836	7.4
Leópolis	295	0.1			
SS Amoreira	1238	0.5			
Cambará	2044	0.8	RS 19 (21)	13,018	4.4
Maripá	997	0.4	RS 20 (17)	45,178	15.4
Total	271,114	100	Total	293,940	100.0

**Table 4:** Dengue incidence rates per 100,000 inhabitants and rate ratios, according to vaccination participation group and age group, in the 10 health regions of Paraná state, Brazil, during the pre-vaccination (2008–2015) and post-vaccination (2019–2022) periods.

Period	Group	Age Group		
		Within the Eligible Age Range for Vaccination	Below the Eligible Age Range for Vaccination	Above the Eligible Age Range for Vaccination
**Pre-vaccination period**	**Participant**	17	9.4	14
	**Non-participant**	9.4	6.3	10
**Post-vaccination period**	**Participant**	40.3	30.6	43.2
	**Non-participant**	41.7	28.7	44.6
RR * **(Post/Pre)**	**Participant**	2.4	3.3	3.1
	**Non-participant**	4.4	4.6	4.5
* Rate Ratio				

## Data Availability

The datasets used and/or analyzed during the current study are available from the corresponding author upon reasonable request.

## Supplementary Materials

The following supporting information can be downloaded at: https://www.mdpi.com/article/10.3390/tropicalmed11010011/s1, Material S1: Estimated populations for three age groups (below vaccination range, within vaccination range, above vaccination range) by municipality and year; Material S2: Methodology for creating the climate variable; Material S3: Estimates and 95% credibility intervals of posterior of model effects and observed versus predicted series; Material S4: Time series with results of prediction scenarios.

## Abbreviations

The following abbreviations are used in this manuscript:

DENV-1	Dengue Virus serotype 1
DENV-2	Dengue Virus serotype 2
DENV-3	Dengue Virus serotype 3
DENV-4	Dengue Virus serotype 4
CYD-TDV	Chimeric Yellow Fever Virus-Dengue Tetravalent Vaccine
ANVISA	Agência Nacional de Vigilância Sanitária
SINAN	Sistema de Informação de Agravos de Notificação
RS	Regional de Saúde
SESA	Secretaria de Estado da Saúde
PR	Paraná
IBGE	Instituto Brasileiro de Geografia e Estatística
VC	Vaccination Coverage
CCD	Central Composite Design
RR	Rate Ratio
CI	Confidence Interval/Credible Interval
HR	Hazard Ratio
