# Supplementary material for: Dengue Incidence Following Mass Vaccination: An Interrupted Time Series Study in Paraná, Brazil
Source: Trop Med Infect Dis. 2025 Dec 30;11(1):11. doi: 10.3390/tropicalmed11010011 (PMC12846613; doi:10.3390/tropicalmed11010011)
Supplement: Supplementary file 1 [file tropicalmed-11-00011-s001.zip › Supplementary Material 1.pdf]

Supplementary Material 1 – Estimated populations for three age groups (below vaccination range, within vaccination range, above vaccination range) by municipality and year.

Table 1: Estimated population (in thousand inhabitants) aged below the vaccination age group, by municipality and health region, Paraná, 2008 to 2022.

Municipality/ Health Region	YEAR														
	2008	2009	2010	2011	2012	2013	2014	2015	2016	2017	2018	2019	2020	2021	2022
Assaí	2.0	2.0	2.0	2.0	2.0	2.0	2.0	2.0	2.0	2.2	2.3	2.6	2.8	3.0	3.2
BV Aparecida	2.1	2.1	2.1	2.0	2.0	2.1	2.1	2.1	2.1	2.2	2.3	2.4	2.5	2.6	2.7
BV Paraíso	3.2	3.2	3.1	3.1	3.1	3.2	3.2	3.2	3.2	3.5	3.7	4.0	4.2	4.5	4.8
C Sul	1.0	1.0	1.0	1.0	1.0	1.0	1.0	1.0	1.0	1.1	1.1	1.2	1.3	1.3	1.4
Cambará	5.3	5.3	5.1	5.1	5.1	5.3	5.3	5.3	5.4	5.9	6.3	6.8	7.1	7.6	8.1
Cambé	21.2	21.4	21.3	21.4	21.6	22.5	22.7	22.8	23.0	25.1	26.8	28.8	30.7	32.8	35.0
F Iguaçu	81.2	82.7	65.2	65.1	65.1	67.1	67.1	67.1	67.2	72.6	76.2	81.2	85.9	90.5	96.0
Ibiporã	10.4	10.5	10.6	10.7	10.8	11.3	11.4	11.5	11.6	12.7	13.7	14.8	15.9	17.0	18.1
Iguaraçu	0.8	0.8	0.8	0.8	0.8	0.9	0.9	0.9	0.9	1.0	1.1	1.2	1.3	1.4	1.5
Itambaracá	1.6	1.6	1.5	1.5	1.5	1.5	1.5	1.5	1.5	1.6	1.7	1.8	1.9	2.0	2.1
Jataizinho	2.9	2.9	3.0	3.0	3.0	3.1	3.1	3.1	3.1	3.4	3.6	3.9	4.1	4.3	4.6
Leópolis	0.9	0.9	0.9	0.9	0.9	0.9	0.9	0.9	0.9	0.9	1.0	1.0	1.1	1.1	1.2
Londrina	105.0	106.1	105.3	106.2	107.1	111.7	112.8	113.9	115.0	125.2	135.4	146.0	157.0	168.4	180.6
M Melo	0.8	0.8	0.7	0.8	0.8	0.8	0.8	0.8	0.8	0.9	1.0	1.0	1.1	1.2	1.3
Mandaguari	6.7	6.7	6.7	6.7	6.7	6.9	7.0	7.0	7.0	7.7	8.2	8.8	9.4	9.9	10.6
Marialva	6.5	6.5	6.6	6.7	6.7	7.0	7.1	7.1	7.2	7.8	8.4	9.1	9.8	10.5	11.2
Maringá	61.8	62.5	66.5	67.5	68.5	71.9	73.0	74.1	75.1	82.1	90.6	98.8	107.7	117.4	127.3
Maripá	1.1	1.1	1.1	1.1	1.1	1.1	1.1	1.1	1.1	1.2	1.3	1.4	1.4	1.5	1.6
Paiçandu	8.3	8.4	8.2	8.3	8.3	8.7	8.8	8.9	9.0	9.9	10.8	11.7	12.6	13.5	14.4
Paranaguá	20.6	20.8	20.9	21.0	21.2	22.0	22.2	22.4	22.6	25.5	28.7	31.8	35.1	38.5	42.2
Porecatu	3.0	2.9	2.9	2.9	2.9	2.9	2.9	2.9	2.9	3.1	3.2	3.4	3.6	3.7	4.0
Santa Fé	2.1	2.1	2.1	2.1	2.1	2.2	2.3	2.3	2.3	2.6	2.8	3.0	3.3	3.5	3.8
Sarandi	20.1	20.4	19.9	20.1	20.3	21.3	21.5	21.7	22.0	24.5	26.6	28.7	30.7	32.8	34.9
Sertanópolis	3.3	3.3	3.2	3.2	3.2	3.3	3.4	3.4	3.4	3.7	4.0	4.2	4.5	4.8	5.1
SI Ivaí	1.7	1.7	1.7	1.7	1.7	1.8	1.8	1.8	1.8	1.9	2.0	2.1	2.3	2.4	2.5
SJ Ivaí	1.1	1.1	1.1	1.1	1.1	1.1	1.1	1.1	1.1	1.2	1.3	1.3	1.4	1.5	1.6
SM Iguaçu	6.2	6.2	6.0	6.1	6.1	6.3	6.3	6.4	6.4	7.0	7.5	8.1	8.7	9.2	9.8
SS Amoreira	2.0	2.1	2.0	2.0	2.0	2.0	2.0	2.0	2.0	2.2	2.4	2.6	2.7	2.9	3.1
ST Itaipu	4.8	4.9	5.0	5.0	5.0	5.3	5.3	5.4	5.4	5.9	6.4	7.0	7.6	8.1	8.6
Tapira	1.2	1.2	1.2	1.2	1.2	1.2	1.2	1.2	1.2	1.3	1.3	1.4	1.5	1.6	1.7
HR 01 (6)	16.1	16.2	17.2	17.3	17.5	18.3	18.5	18.7	18.9	21.4	24.3	27.3	30.4	33.6	36.9
HR 09 (6)	18.9	19.0	19.3	19.4	19.5	20.3	20.5	20.7	20.8	22.8	24.8	26.7	28.6	30.6	32.6
HR 10 (24)	117.6	118.6	116.0	116.7	117.3	121.9	122.8	123.6	124.4	135.8	146.2	157.2	168.5	179.5	191.3
HR 12 (20)	54.0	54.1	54.0	54.1	54.2	56.1	56.3	56.5	56.6	61.6	65.5	70.4	75.4	80.2	85.6
HR 14 (26)	54.5	54.7	54.8	55.0	55.2	57.2	57.5	57.8	58.0	63.5	68.0	73.0	78.0	82.8	88.1
HR 15 (21)	34.7	34.8	35.1	35.3	35.4	36.7	36.9	37.1	37.3	40.9	44.0	47.4	50.6	53.9	57.5
HR 17 (13)	31.3	31.5	31.9	32.1	32.3	33.6	33.9	34.2	34.5	37.6	40.5	43.7	46.8	49.9	53.2
HR 18 (18)	45.9	45.8	44.6	44.5	44.3	45.6	45.5	45.4	45.3	49.1	51.5	55.0	58.1	61.2	65.0
HR 19 (21)	58.2	58.3	58.0	58.1	58.2	60.1	60.3	60.5	60.6	65.8	69.8	74.5	79.1	83.4	88.5
HR 20 (17)	74.8	75.5	75.8	76.4	77.0	80.2	81.0	81.7	82.4	90.1	97.5	105.4	113.5	121.6	129.8

Table 2: Estimated population (in thousand inhabitants) within the vaccination age group, by municipality and health region, Paraná, 2008 to 2022.

Municipality/Health Region	YEAR														
	**2008**	**2009**	**2010**	**2011**	**2012**	**2013**	**2014**	**2015**	**2016**	**2017**	**2018**	**2019**	**2020**	**2021**	**2022**
**Assaí**	9.0	8.9	9.0	8.9	8.9	9.0	9.0	8.9	8.9	8.8	8.3	8.2	8.1	7.9	7.9
**BV Aparecida**	1.5	1.5	1.5	1.5	1.5	1.5	1.5	1.5	1.5	1.4	1.3	1.3	1.3	1.3	1.3
**BV Paraíso**	3.2	3.2	3.1	3.1	3.1	3.2	3.2	3.3	3.3	3.2	3.1	3.1	3.1	3.0	3.0
**C Sul**	1.0	1.0	1.0	1.0	1.0	1.0	1.0	1.0	1.0	1.0	1.0	0.9	0.9	0.9	0.9
**Cambará**	5.3	5.3	5.1	5.1	5.1	5.3	5.3	5.3	5.3	5.3	5.2	5.1	5.2	5.2	5.2
**Cambé**	21.1	21.3	21.2	21.3	21.4	22.4	22.5	22.7	22.9	22.9	23.0	23.2	23.4	23.5	23.7
**F Iguaçu**	75.3	76.7	60.4	60.4	60.3	62.2	62.2	62.2	62.3	61.4	59.5	58.7	58.1	57.7	58.0
**Ibiporã**	10.1	10.2	10.4	10.5	10.6	11.1	11.2	11.3	11.4	11.4	11.6	11.7	11.8	11.9	12.0
**Iguaraçu**	0.9	0.9	0.9	0.9	0.9	1.0	1.0	1.0	1.0	1.0	1.0	1.0	1.0	0.9	0.9
**Itambaracá**	1.4	1.4	1.4	1.4	1.4	1.4	1.4	1.4	1.4	1.4	1.3	1.3	1.2	1.2	1.3
**Jataizinho**	2.6	2.6	2.7	2.7	2.7	2.8	2.8	2.8	2.8	2.8	2.7	2.7	2.6	2.6	2.6
**Leópolis**	0.9	0.9	0.9	0.9	0.9	0.9	0.9	0.9	0.9	0.8	0.8	0.8	0.8	0.8	0.8
**Londrina**	113.1	114.3	113.4	114.5	115.5	120.3	121.6	122.7	123.9	125.6	127.7	129.1	130.2	130.9	131.3
**M Melo**	0.8	0.8	0.8	0.8	0.8	0.8	0.8	0.8	0.8	0.8	0.8	0.8	0.7	0.7	0.7
**Mandaguari**	7.0	7.1	7.0	7.0	7.0	7.3	7.3	7.3	7.4	7.3	7.2	7.2	7.2	7.2	7.2
**Marialva**	6.9	6.9	7.1	7.1	7.2	7.5	7.5	7.6	7.7	7.7	7.8	7.8	7.8	7.8	7.8
**Maringá**	78.3	79.3	84.4	85.6	86.8	91.1	92.6	93.9	95.2	97.1	100.7	102.4	103.4	104.0	103.3
**Maripá**	1.1	1.1	1.1	1.1	1.1	1.2	1.2	1.2	1.2	1.1	1.1	1.1	1.1	1.1	1.1
**Paiçandu**	8.5	8.6	8.4	8.5	8.6	9.0	9.1	9.2	9.3	9.3	9.5	9.5	9.5	9.6	9.6
**Paranaguá**	84.0	84.6	85.0	85.6	86.2	89.7	90.5	91.2	91.9	91.9	91.2	91.0	90.8	90.4	89.6
**Porecatu**	3.0	3.0	3.0	2.9	2.9	3.0	3.0	2.9	2.9	2.8	2.6	2.5	2.5	2.4	2.4
**Santa Fé**	2.2	2.3	2.3	2.3	2.3	2.4	2.5	2.5	2.5	2.5	2.5	2.6	2.6	2.6	2.6
**Sarandi**	19.5	19.7	19.3	19.5	19.7	20.6	20.8	21.1	21.3	21.7	22.0	22.0	22.1	22.3	22.3
**Sertanópolis**	3.4	3.4	3.3	3.3	3.3	3.5	3.5	3.5	3.5	3.5	3.4	3.4	3.4	3.3	3.3
**SI Ivaí**	1.7	1.7	1.7	1.7	1.7	1.8	1.8	1.8	1.8	1.7	1.7	1.6	1.6	1.6	1.6
**SJ Ivaí**	1.0	1.0	1.0	1.0	1.0	1.1	1.1	1.1	1.1	1.1	1.0	1.0	1.0	1.0	1.0
**SM Iguaçu**	6.1	6.1	5.9	6.0	6.0	6.2	6.2	6.3	6.3	6.2	6.0	5.9	5.8	5.8	5.8
**SS Amoreira**	2.0	2.0	1.9	1.9	1.9	2.0	2.0	2.0	2.0	2.0	1.9	1.8	1.8	1.7	1.7
**ST Itaipu**	4.7	4.8	4.8	4.9	4.9	5.1	5.2	5.2	5.3	5.2	5.2	5.1	5.1	5.0	5.1
**Tapira**	1.2	1.2	1.1	1.1	1.1	1.2	1.2	1.2	1.1	1.1	1.1	1.0	1.0	1.0	1.0
**HR 01 (6)**	65.1	65.5	69.8	70.5	71.2	74.3	75.1	75.9	76.7	76.9	77.1	77.1	77.1	77.0	76.5
**HR 09 (6)**	19.1	19.2	19.5	19.7	19.8	20.6	20.8	21.0	21.1	21.0	20.7	20.6	20.6	20.4	20.4
**HR 10 (24)**	118.0	119.0	116.4	117.1	117.8	122.5	123.4	124.3	125.2	124.6	123.8	123.2	122.6	122.3	122.4
**HR 12 (20)**	56.1	56.2	56.2	56.4	56.5	58.4	58.7	58.9	59.1	58.8	57.8	57.3	56.8	56.5	56.5
**HR 14 (26)**	54.1	54.4	54.4	54.6	54.8	56.8	57.1	57.4	57.7	56.8	55.8	55.1	54.7	54.3	54.3
**HR 15 (21)**	36.2	36.3	36.7	36.9	37.0	38.4	38.6	38.8	39.0	38.5	37.8	37.6	37.4	37.3	37.4
**HR 17 (13)**	31.6	31.8	32.2	32.4	32.6	33.9	34.2	34.5	34.7	34.7	34.6	34.5	34.5	34.5	34.6
**HR 18 (18)**	44.6	44.5	43.3	43.2	43.1	44.3	44.2	44.1	44.1	43.2	41.3	40.4	39.7	39.2	39.3
**HR 19 (21)**	54.5	54.6	54.2	54.3	54.4	56.2	56.4	56.6	56.7	56.0	54.6	53.9	53.4	53.3	53.4
**HR 20 (17)**	78.1	78.8	79.2	79.9	80.5	83.9	84.7	85.4	86.2	86.2	86.4	86.3	85.9	85.8	85.8

Table 3: Estimated population (in thousand inhabitants) above the vaccination age group, by municipality and health region, Paraná, 2008 to 2022

Municipality/Health Region	YEAR														
	2008	2009	2010	2011	2012	2013	2014	2015	2016	2017	2018	2019	2020	2021	2022
Assaí	5.4	5.3	5.4	5.3	5.3	5.4	5.4	5.3	5.3	5.0	4.6	4.4	4.1	3.9	3.8
BV Aparecida	4.4	4.4	4.4	4.3	4.3	4.4	4.4	4.4	4.4	4.3	4.0	3.9	3.7	3.6	3.6
BV Paraíso	9.0	9.1	8.8	8.8	8.8	9.1	9.1	9.1	9.1	8.9	8.6	8.3	8.1	7.9	7.8
C Sul	2.6	2.6	2.6	2.6	2.6	2.6	2.6	2.6	2.6	2.6	2.4	2.3	2.3	2.2	2.2
Cambará	14.3	14.5	13.8	13.8	13.9	14.4	14.4	14.5	14.6	14.2	13.8	13.5	13.1	12.8	12.6
Cambé	54.2	54.6	54.3	54.7	55.0	57.4	57.8	58.3	58.7	57.4	55.8	54.5	53.2	51.8	50.5
F Iguaçu	162.6	165.7	130.5	130.4	130.3	134.3	134.3	134.4	134.5	130.1	123.1	118.6	114.2	109.8	106.5
Ibiporã	26.5	26.8	27.2	27.4	27.7	28.9	29.2	29.5	29.8	29.2	28.7	28.1	27.5	26.8	26.1
Iguaraçu	2.2	2.2	2.2	2.2	2.3	2.4	2.4	2.4	2.4	2.4	2.3	2.3	2.2	2.2	2.1
Itambaracá	4.1	4.1	3.9	3.9	3.9	4.0	3.9	3.9	3.9	3.8	3.6	3.5	3.4	3.3	3.2
Jataizinho	6.1	6.1	6.3	6.3	6.3	6.5	6.6	6.6	6.6	6.4	6.2	6.0	5.9	5.7	5.6
Leópolis	2.5	2.5	2.4	2.4	2.4	2.4	2.4	2.4	2.4	2.3	2.2	2.1	2.1	2.0	1.9
Londrina	287.1	290.3	288.0	290.6	293.1	305.5	308.6	311.6	314.5	307.6	300.8	294.6	288.2	281.5	274.7
M Melo	2.2	2.2	2.1	2.2	2.2	2.3	2.3	2.3	2.3	2.3	2.2	2.2	2.2	2.1	2.1
Mandaguari	19.2	19.3	19.0	19.1	19.1	19.8	19.9	20.0	20.1	19.6	18.9	18.4	18.0	17.5	17.2
Marialva	17.8	18.0	18.3	18.4	18.6	19.3	19.5	19.7	19.8	19.4	18.9	18.6	18.2	17.8	17.4
Maringá	191.4	193.7	206.2	209.2	212.1	222.7	226.2	229.5	232.7	227.5	225.7	222.5	219.0	215.1	210.2
Maripá	3.5	3.4	3.5	3.4	3.4	3.5	3.5	3.5	3.5	3.4	3.3	3.2	3.1	3.0	2.9
Paiçandu	19.6	19.8	19.3	19.6	19.8	20.7	20.9	21.1	21.4	20.9	20.5	20.1	19.7	19.2	18.7
Paranaguá	34.1	34.4	34.6	34.8	35.1	36.5	36.8	37.1	37.4	35.7	33.8	32.1	30.3	28.5	27.2
Porecatu	8.4	8.3	8.3	8.2	8.1	8.3	8.2	8.2	8.1	7.8	7.3	7.0	6.7	6.4	6.3
Santa Fé	5.9	6.0	6.1	6.1	6.2	6.5	6.6	6.6	6.7	6.6	6.5	6.4	6.3	6.2	6.1
Sarandi	43.9	44.5	43.6	44.1	44.5	46.5	47.0	47.6	48.1	47.9	47.0	46.0	45.0	43.9	42.7
Sertanópolis	9.3	9.4	9.1	9.1	9.1	9.5	9.5	9.5	9.6	9.3	9.0	8.8	8.5	8.3	8.2
SI Ivaí	5.2	5.2	5.3	5.2	5.2	5.4	5.4	5.3	5.3	5.2	4.9	4.8	4.7	4.5	4.5
SJ Ivaí	3.3	3.3	3.4	3.4	3.4	3.5	3.5	3.5	3.5	3.4	3.3	3.2	3.1	3.0	3.0
SM Iguaçu	14.1	14.2	13.8	13.8	13.9	14.4	14.5	14.6	14.6	14.3	13.8	13.4	13.1	12.7	12.4
SS Amoreira	4.9	4.9	4.7	4.7	4.7	4.9	4.9	4.9	4.9	4.8	4.6	4.5	4.3	4.2	4.1
ST Itaipu	10.8	10.9	11.1	11.2	11.3	11.7	11.9	12.0	12.1	11.8	11.6	11.3	11.1	10.8	10.6
Tapira	3.5	3.5	3.5	3.5	3.4	3.5	3.5	3.5	3.5	3.4	3.2	3.1	3.0	2.9	2.9
HR 01 (6)	35.3	35.5	38.0	38.3	38.7	40.4	40.9	41.3	41.8	40.4	39.1	37.6	36.2	34.6	33.2
HR 09 (6)	46.3	46.6	47.3	47.6	48.0	49.9	50.3	50.7	51.1	50.0	48.7	47.6	46.6	45.4	44.4
HR 10 (24)	270.8	273.0	267.1	268.6	270.1	280.5	282.4	284.2	286.1	278.7	269.5	262.6	255.6	248.3	242.0
HR 12 (20)	148.8	149.1	149.0	149.4	149.7	154.8	155.3	155.8	156.3	152.6	146.8	143.1	139.3	135.5	132.7
HR 14 (26)	137.4	137.9	138.0	138.6	139.1	144.1	144.9	145.6	146.2	142.8	138.0	134.8	131.5	128.1	125.4
HR 15 (21)	96.3	96.6	97.5	97.9	98.3	101.9	102.4	102.9	103.4	101.1	97.8	95.5	93.2	90.9	89.1
HR 17 (13)	81.0	81.5	82.4	82.9	83.5	86.8	87.5	88.2	88.8	86.9	84.7	82.9	81.0	79.1	77.4
HR 18 (18)	122.0	121.8	118.5	118.2	117.9	121.1	120.9	120.7	120.5	117.3	111.2	107.8	104.5	101.3	99.4
HR 19 (21)	142.6	142.9	142.0	142.2	142.4	147.2	147.6	148.0	148.4	144.7	138.8	135.2	131.6	127.9	125.3
HR 20 (17)	195.4	197.1	197.9	199.5	201.0	209.3	211.2	213.0	214.8	210.3	205.3	201.1	196.7	192.2	188.0
