# Supplementary material for: Dengue Incidence Following Mass Vaccination: An Interrupted Time Series Study in Paraná, Brazil
Source: Trop Med Infect Dis. 2025 Dec 30;11(1):11. doi: 10.3390/tropicalmed11010011 (PMC12846613; doi:10.3390/tropicalmed11010011)
Supplement: Supplementary file 1 [file tropicalmed-11-00011-s001.zip › Supplementary Material 2.pdf]

Supplementary Material 2 – Methodology for creating the climate variable

The National Institute of Meteorology - INMET provides Brazilian climatological data via the website: https://portal.inmet.gov.br. We consider data from the network of automatic stations. For these stations, time series with hourly resolution are available for the following variables:

- TOTAL RAINFALL, TIME (mm)
- MAX. ATMOSPHERIC PRESSURE AT THE PREVIOUS TIME. (mB)
- GLOBAL RADIATION (KJ/m²)
- DEW POINT TEMPERATURE (°C)
- MINIMUM TEMPERATURE AT THE PREVIOUS HOUR. (°C)
- DEW TEMPERATURE MIN. AT THE PREVIOUS HOUR. (°C)
- REL. HUMIDITY MIN. AT THE PREVIOUS HOUR. (%)
- WIND DIRECTION (RADIAN DEGREES)
- WIND SPEED (m/s)

The only climate variable included in the model to estimate vaccine effectiveness was a function of the minimum temperature at the previous hour [12].

Data from 26 stations located in the state and another 33 stations, located within one degree radius around Paraná, contributed to the calculations (symbols colored in blue on the map in Figure 1).

Figure 1: Geographical distribution of meteorological stations in the state of Paraná, 2022.

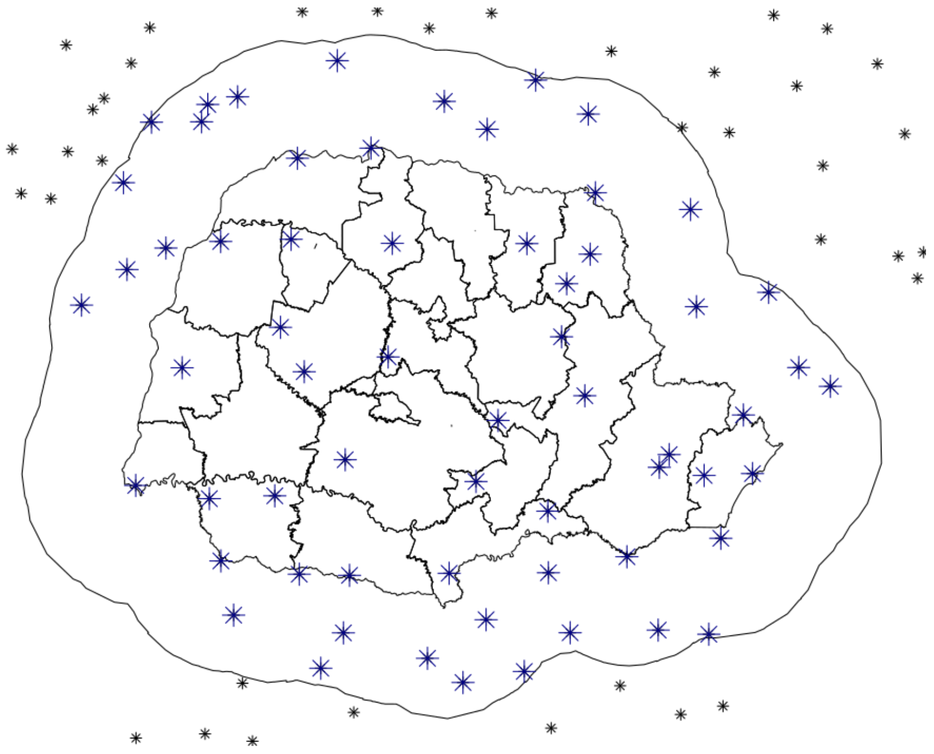

Figure 2 shows the series of minimum temperature solely for the year 2020 to facilitate visualization. A seasonal pattern is observed when the minimum temperature value is lower in the middle of the year.

Figure 2: Minimum temperatures recorded in the year 2020 at stations in Paraná and those located within a radius of one degree around Paraná.

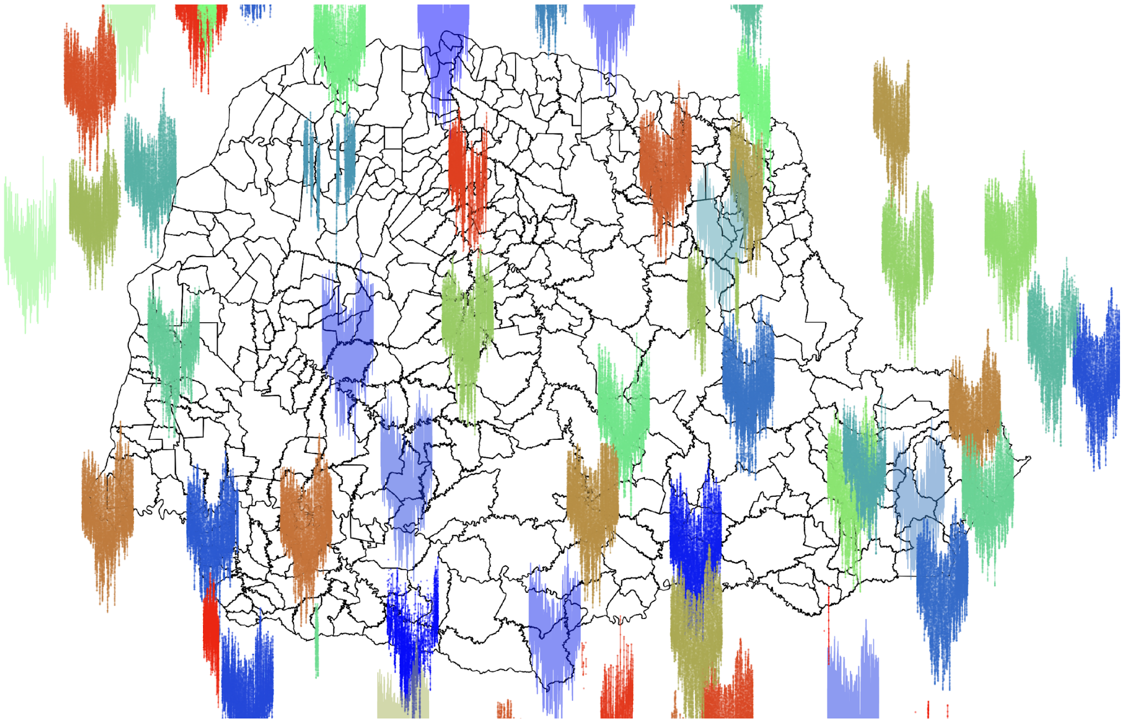

Figure 3 considers the series for the first 7 days of January 2020 only. It shows the daily minimum temperature pattern in each of the time series. The dashed line marks the temperature threshold of 21°C.

Figure 3: Minimum temperatures recorded in the first week of 2020 at weather stations in Paraná and those located within a radius of one degree around Paraná.

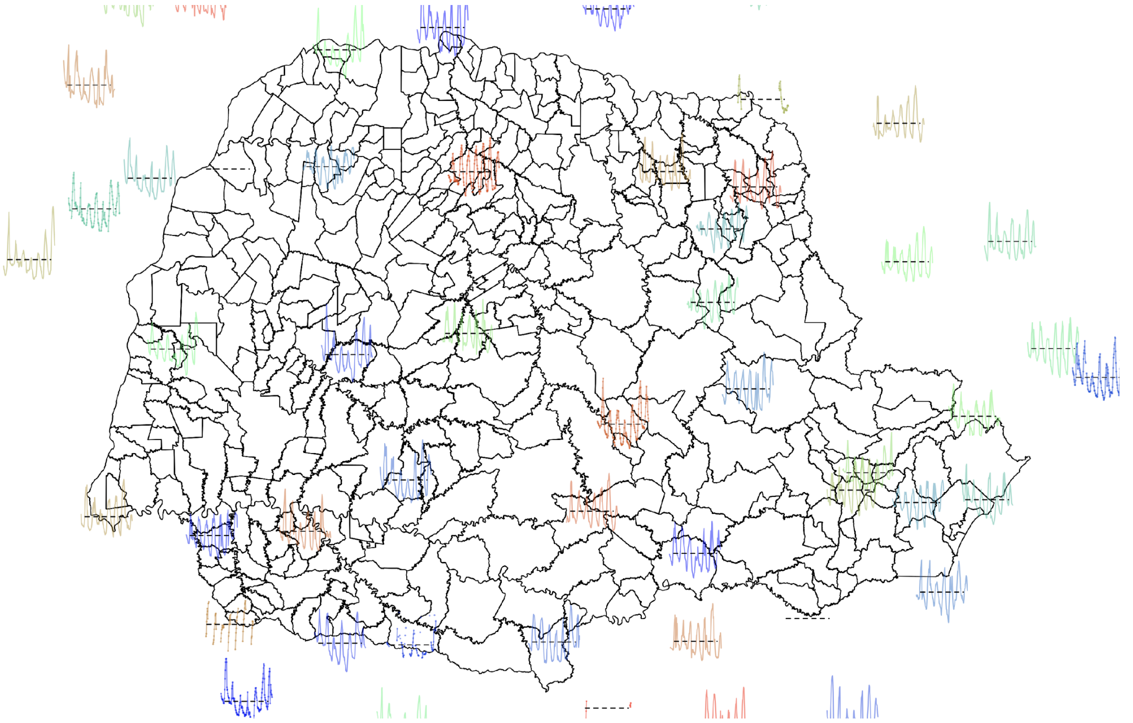

For the definition of the covariate based on the minimum temperature, we considered minima in each hour and calculated the proportion of values above 21ºC [13-17]. For example, within one week, we have 168 values and the covariate is defined as the number of hours that the minimum temperature was above 21ºC divided by 168. Thus, we have the covariate as the proportion of time when the minimum temperature was above 21ºC. The climate variable effectively considered in the modeling of dengue incidence considered a lag period of 4 weeks (672 hours) between 9 and 12 weeks prior to the outcome.

Space-time interpolation

A space-time stochastic model [18] was adopted to make predictions in the 40 places of interest considering the climate data observed in the 59 meteorological stations. Based on this model, we predicted the time series for each of the 202 municipalities, which were then aggregated into predicted time series for the ten groups of municipalities in each health region (Figure 4).

Figure 4: Estimated hourly time series for 30 municipalities in the survey for the first week of 2021.

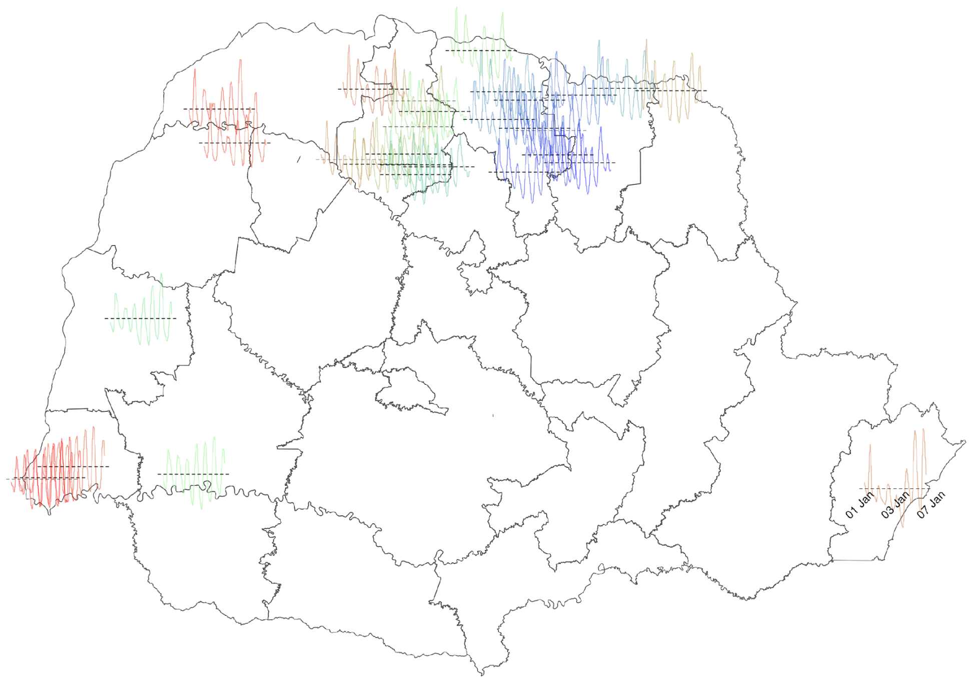

The model for the time series of hourly minimum temperatures, $x_{i,t}$, assumes two space-time effects as specified below

$$x_{i,t} = a + u_{i,t} + v_{i,t} + e_{i,t}$$

u is a spatially correlated and temporally independent space-time process, v is a space-time process correlated in space and time.

The process u aims to capture fluctuations between different months while the process v aims to capture hourly fluctuations. The spatial dependence in each of these processes is considered by assuming a Matérn covariance structure, with a fixed smoothness parameter $\nu = 1$, specified via a stochastic differential equations approach [18,19]. In this approach, different triangulations were considered, a denser one for u and a less dense one for v (Figure 5).

Figure 5: Meshes of triangulations for the space-time model.

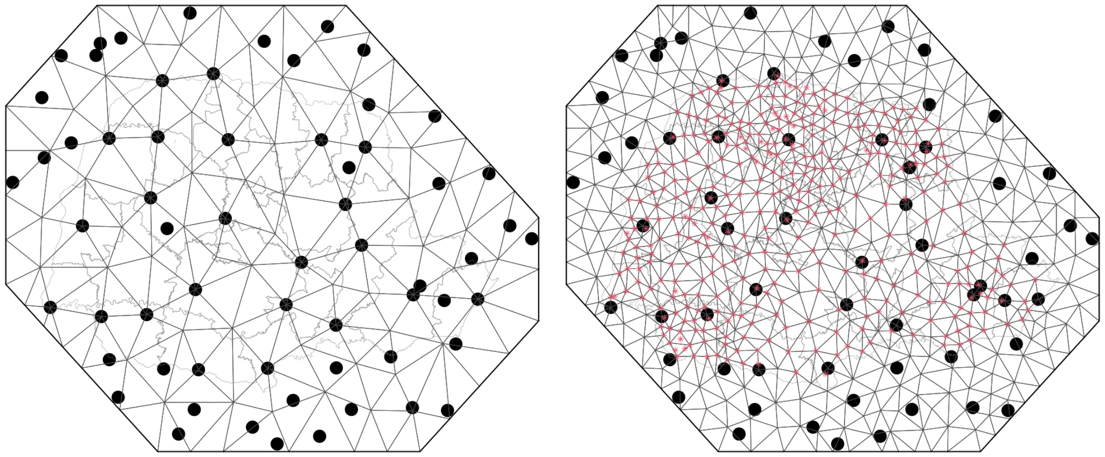

Legend: Sparse triangulations (on the left) and dense triangulations (on the right). The black dots represent the locations of weather stations and the red dots are the centroids of the municipalities in the state of Paraná.

We assumed the following distribution for u to the U grid points for each month,

$$u_{\{1,\dots,U\},m} \sim N\left(0, Q^{-1}(\sigma_u^2, \kappa_u)\right)$$

where σ_u^2 and κ_u are parameters of the Matérn covariance function. For the process v, we considered a model of the form

$$v_k = \phi_1 v_{k-1} + \phi_2 v_{k-2} + w_k$$

where k is the time index for each month and w_k is spatially correlated such that

$$w_k \sim N\left(0, Q^{-1}(\sigma_v^2, \kappa_v)\right)$$

with ϕ_1 and ϕ_2 parameters from a second order autoregressive process. We considered a temporal resolution of two hours, i.e., k such that within a 31 days month, $k = 1,\dots,372$.

Thus, we define the projection matrices M and A such that M projects the process u within each month to the hour t and A projects the process v at every two hours to t, and for each grid to the locations of minimum temperature. We write the model in vector form as

$$x = a + Mu + Av + e$$

We decided to set the parameters κ_u and κ_v such that the practical ranges, are equal to 3 and 1.5, respectively. Furthermore, we set the parameters ϕ_1 and ϕ_2 such that the first and second order partial autocorrelations are equal to 0.97 and -0.6.

We consider hourly data from the year 2017 to the year 2022. Due to the high temporal resolution, we have time series for a total of 134376 hours. We consider the estimation of the model specified above year by year. The goodness of fit of a linear model can be considered in several ways. One is by looking at the variance of the error term in relation to the total variance of the data. This ratio was around 0.15.

The results of the space-time modeling of the climate data were summarized in the calculation of the conditional expectation in each time and place of the research.

The proportion of time in each epidemiological week with a minimum temperature above 21 degrees Celsius in each location was obtained from these hourly series. In the 10 groups of municipalities, we considered the average of the result in each of the municipalities that make up each group.

We included the time series of data available at some of the stations, as well as the value estimated by the model. We have not included them all due to space limitations.

Example of the hourly minimum temperature time series for one of the meteorological stations considered in this study.

The dots represent data, with several periods missing data. The estimates obtained by the climate model are superimposed in red lines. Graphs allow you to visually check the fit of the model to the data.

Observing these time series and considering the horizontal dashed line at the value of 21 degrees Celsius, it is possible to visualize the evolution of this variable over time. The derived variable, proportion of time in which the minimum temperature was above 21 degrees Celsius, was obtained from the estimated value in each hour.

Figure 6: Minimum hourly temperature at the meteorological station located in Cidade Gaúcha, Paraná, 2009.

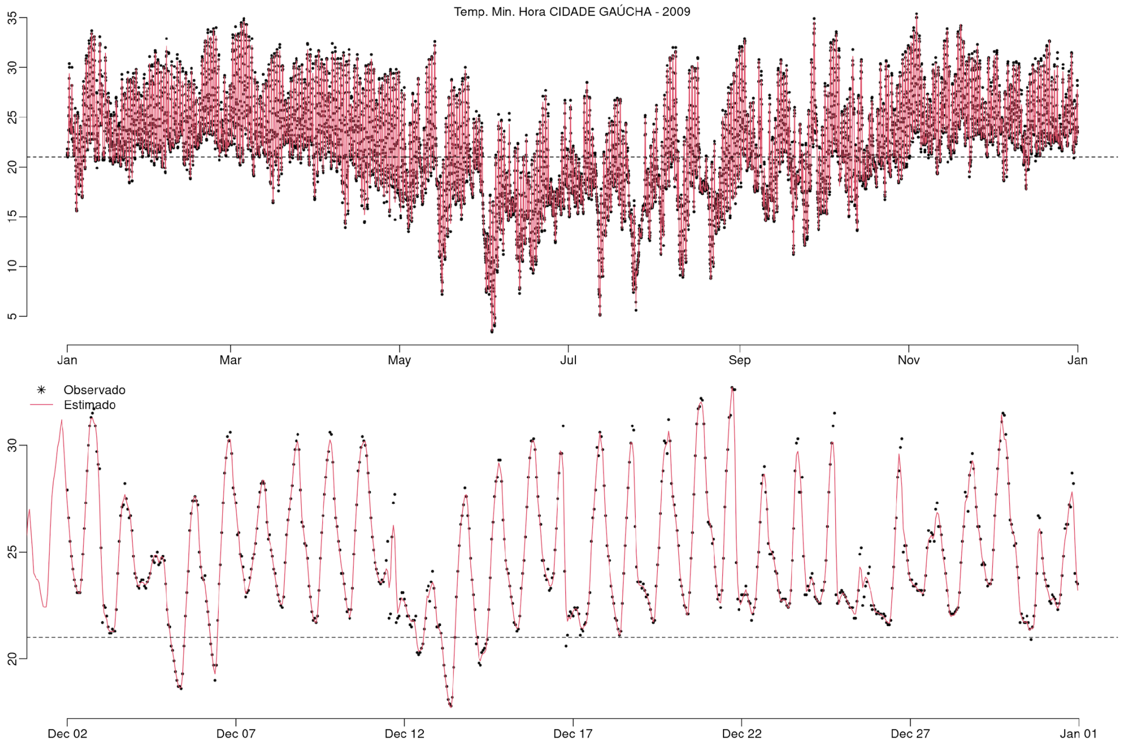

Legend: Observed (black dots) and estimated (red line) minimum hourly temperature for the year of 2009 (top panel); and a zoom view of December 2009 (bottom panel). Dashed line indicates de 21 degrees Celsius threshold.

Figure 7: Minimum hourly temperature at the meteorological station located in Cidade Gaúcha, Paraná, 2011.

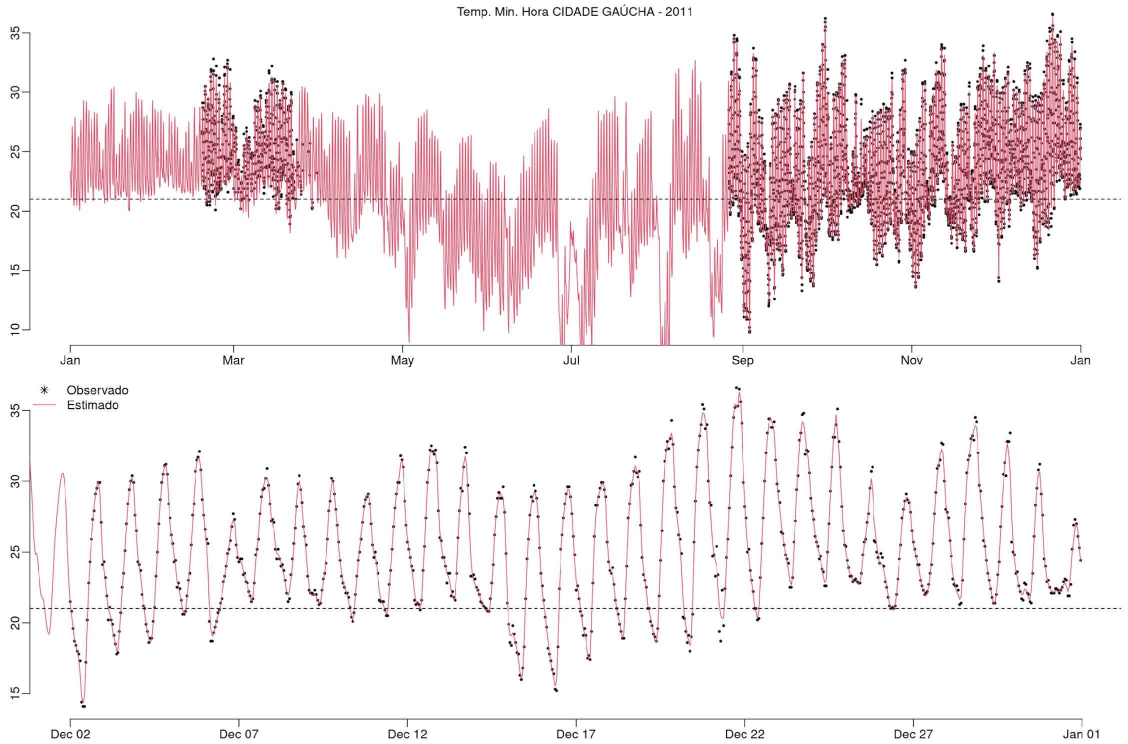

Legend: Observed (black dots) and estimated (red line) minimum hourly temperature for the year of 2011 (top panel); and a zoom view of December 2011 (bottom panel). Dashed line indicates de 21 degrees Celsius threshold.

Figure 8: Minimum hourly temperature at the meteorological station located in Foz do Iguaçu, Paraná, 2010.

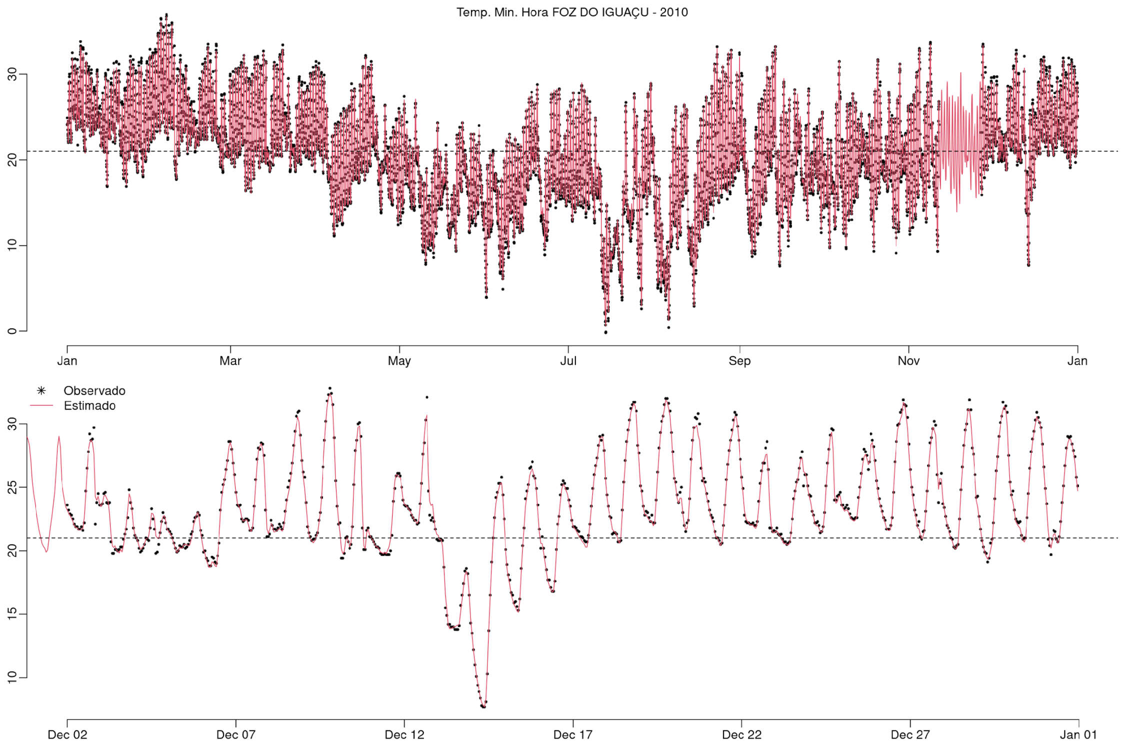

Legend: Observed (black dots) and estimated (red line) minimum hourly temperature for the year of 2010 (top panel); and a zoom view of December 2010 (bottom panel). Dashed line indicates de 21 degrees Celsius threshold.

Figure 9: Minimum hourly temperature at the meteorological station located in Foz do Iguaçu, Paraná, 2021.

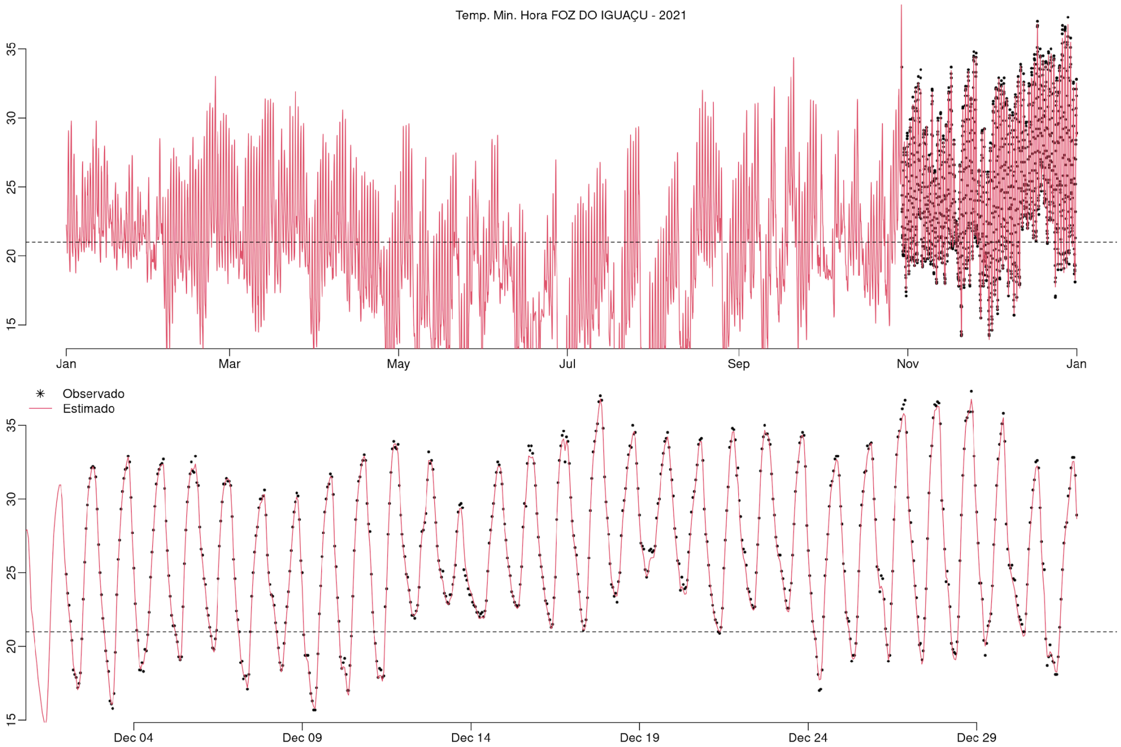

Legend: Observed (black dots) and estimated (red line) minimum hourly temperature for the year of 2021 (top panel); and a zoom view of December 2021 (bottom panel). Dashed line indicates de 21 degrees Celsius threshold.
