# Supplementary material for: Dengue Incidence Following Mass Vaccination: An Interrupted Time Series Study in Paraná, Brazil
Source: Trop Med Infect Dis. 2025 Dec 30;11(1):11. doi: 10.3390/tropicalmed11010011 (PMC12846613; doi:10.3390/tropicalmed11010011)

Supplementary Material 3 - Estimates and 95% credibility intervals of posterior of model effects and observed versus predicted series

The statistical model was adjusted for the time series, y_{itk}, with t indicating the week t, $t = 1$ is the first epidemiological week of 2008 and $t = 766$ is the 37th epidemiological week of 2022, and $k = 1{,}2{,}3$, indicates the age range (below, within and above vaccine range).

The expected number of cases for each location, age group and year to be considered in the model is then given by

$$E_{itk} = m_0\, P_{ika}/n_a$$

where P_{ika} is the population at location i, age range k and year a, which is repeated for every epidemiological week t within year a, and n_a is the number of epidemiological weeks within year a; and m_0 is the gross annual rate obtained from the absolute incidence in the ten regions in the whole period, calculated as

$$m_0 = \frac{\sum_{i=1}^{40}\sum_{a=1}^{15}\sum_{k=1}^{3} y_{ika}}{\sum_{i=1}^{40}\sum_{a=1}^{15}\sum_{k=1}^{3} P_{ika}},$$

where y_{ika} and P_{ika} are the number of dengue cases and population in location i, age group k and year a, respectively, for $i = 1,\ldots,40$, $k = 1{,}2{,}3$, and $a = 1,\ldots,15$ ($a = 1$ is the year 2008).

A Poisson probability distribution was assumed for y_{itk}

$$y_{itk} \sim Poisson(E_{itk}\lambda_{itk})$$

where λ_{itk} is the hazard ratio for location i, at time t and age group k relative to the anual rate of the ten health regions under study.

It is assumed that the linear predictor log(λ_{itk}) is a function of various terms or components, as specified below:

$$log(\lambda_{itk}) = \alpha_i + \delta_k I(Agegroup_{ik}) + \beta_1 Serotype_{1i} + \beta_2 Serotype_{2i} + \beta_3 Serotype_{3i} + \beta_4 Serotype_{4i} + \beta_5 Climate_{it} + \beta_6 VC_{it} + r_{itk}$$ where

- α_i logarithm of the hazard ratio at location i in the vaccine age group,
- δ_k additional (log)hazard ratio of non-vaccine age group *k*,
- $\beta_1,\ldots,\beta_4$ additional (log)hazard ratios due to the presence of serotypes 1 to 4,
- β_5 effect of the climate variable,
- β_6 effect of the vaccine coverage,

- r_{itk} residual temporal effect.

Thus, the specific temporal variation for each location i is given by $\alpha_i + r_{itk}$.

Model estimation method

The Bayesian inference paradigm was used to estimate the model parameters. A Poisson likelihood is considered for the observed data, conditional on the expected number of cases, E_{itk}, and hazard ratio, λ_{itk}. For the effects of the factors in the model, an a priori distribution of N(0,1000) is considered. For the specific temporal random effect of each location, an a priori autoregressive distribution of order 2, AR2, is considered for each location but with common parameters. For the precision parameter, we considered an a priori distribution suggested by [21], with $P(\sigma > 1) = 0.1$. For the partial autocorrelation parameters, we adopted PC priors with parameters and $(3,0.01)$ and $(0.5,0.5)$[22].

For the calculation of the posterior distributions, we considered a Laplace approximation, for the conditional distribution to the hyperparameters and the correction proposed by [23].

Table 1: Estimated coefficients with standard deviations and 95% credibility intervals.

Variables	mean	sd	0.025quant	0.975quant
**Assaí**	-3.468	0.168	-3.799	-3.138
**BV Aparecida**	-4.903	0.231	-5.356	-4.450
**BV Paraíso**	-2.880	0.136	-3.147	-2.613
**C Sul**	-4.051	0.232	-4.506	-3.597
**Cambará**	-4.983	0.203	-5.381	-4.585
**Cambé**	-3.426	0.122	-3.664	-3.187
**F Iguaçu**	-2.754	0.113	-2.974	-2.533
**Ibiporã**	-3.291	0.134	-3.554	-3.027
**Iguaraçu**	-4.649	0.266	-5.172	-4.128
**Itambaracá**	-4.420	0.239	-4.888	-3.952
**Jataizinho**	-1.128	0.118	-1.359	-0.896
**Leópolis**	-5.016	0.303	-5.610	-4.422
**Londrina**	-3.339	0.114	-3.562	-3.116
**M Melo**	-4.640	0.275	-5.179	-4.102
**Mandaguari**	-4.573	0.168	-4.903	-4.244
**Marialva**	-4.431	0.159	-4.743	-4.120
**Maringá**	-4.013	0.104	-4.216	-3.810
**Maripá**	-4.184	0.216	-4.608	-3.760
**Paiçandu**	-4.577	0.158	-4.887	-4.268
**Paranaguá**	-5.620	0.165	-5.943	-5.297
**Porecatu**	-3.182	0.154	-3.485	-2.879
**Santa Fé**	-3.813	0.169	-4.145	-3.482
**Sarandi**	-3.927	0.121	-4.164	-3.690
**Sertanópolis**	-2.755	0.137	-3.024	-2.486
**SI Ivaí**	-3.643	0.189	-4.013	-3.273
**SJ Ivaí**	-5.103	0.284	-5.661	-4.546
**SM Iguaçu**	-4.120	0.171	-4.456	-3.785
**SS Amoreira**	-5.303	0.263	-5.820	-4.787
**ST Itaipu**	-3.078	0.142	-3.357	-2.798
**Tapira**	-3.635	0.176	-3.981	-3.289
**RS 01 (6)**	-5.975	0.185	-6.338	-5.613
**RS 09 (6)**	-3.407	0.131	-3.664	-3.150
**RS 10 (24)**	-4.003	0.113	-4.225	-3.782
**RS 12 (20)**	-3.388	0.096	-3.576	-3.200
**RS 14 (26)**	-3.467	0.107	-3.676	-3.258
**RS 15 (21)**	-3.710	0.110	-3.925	-3.495
**RS 17 (13)**	-2.019	0.112	-2.238	-1.799
**RS 18 (18)**	-4.280	0.125	-4.525	-4.035
**RS 19 (21)**	-4.791	0.119	-5.025	-4.558
**RS 20 (17)**	-4.056	0.108	-4.267	-3.846
**Abaixo vacinal**	-0.475	0.004	-0.483	-0.466
**Acima vacinal**	-0.044	0.004	-0.051	-0.038
**DENV1**	1.252	0.127	1.004	1.501
**DENV2**	0.005	0.087	-0.165	0.175
**DENV3**	-0.299	0.179	-0.649	0.052
**DENV4**	0.051	0.072	-0.091	0.193
**VC**	-0.207	0.023	-0.252	-0.161
**Climate**	1.832	0.095	1.646	2.018

Table 2: Summary of model hyperparameters.

Parameters	mean	sd	0.025quant	0.975quant

Parameters	mean	sd	0.025quant	0.975quant
**Precision for week**	0.200	0.007	0.186	0.213
**PACF1 for week**	0.981	0.001	0.980	0.982
**PACF2 for week**	-0.480	0.012	-0.503	-0.457

Figure 1: Estimated specific temporal variation by municipality and 95% credible intervals.

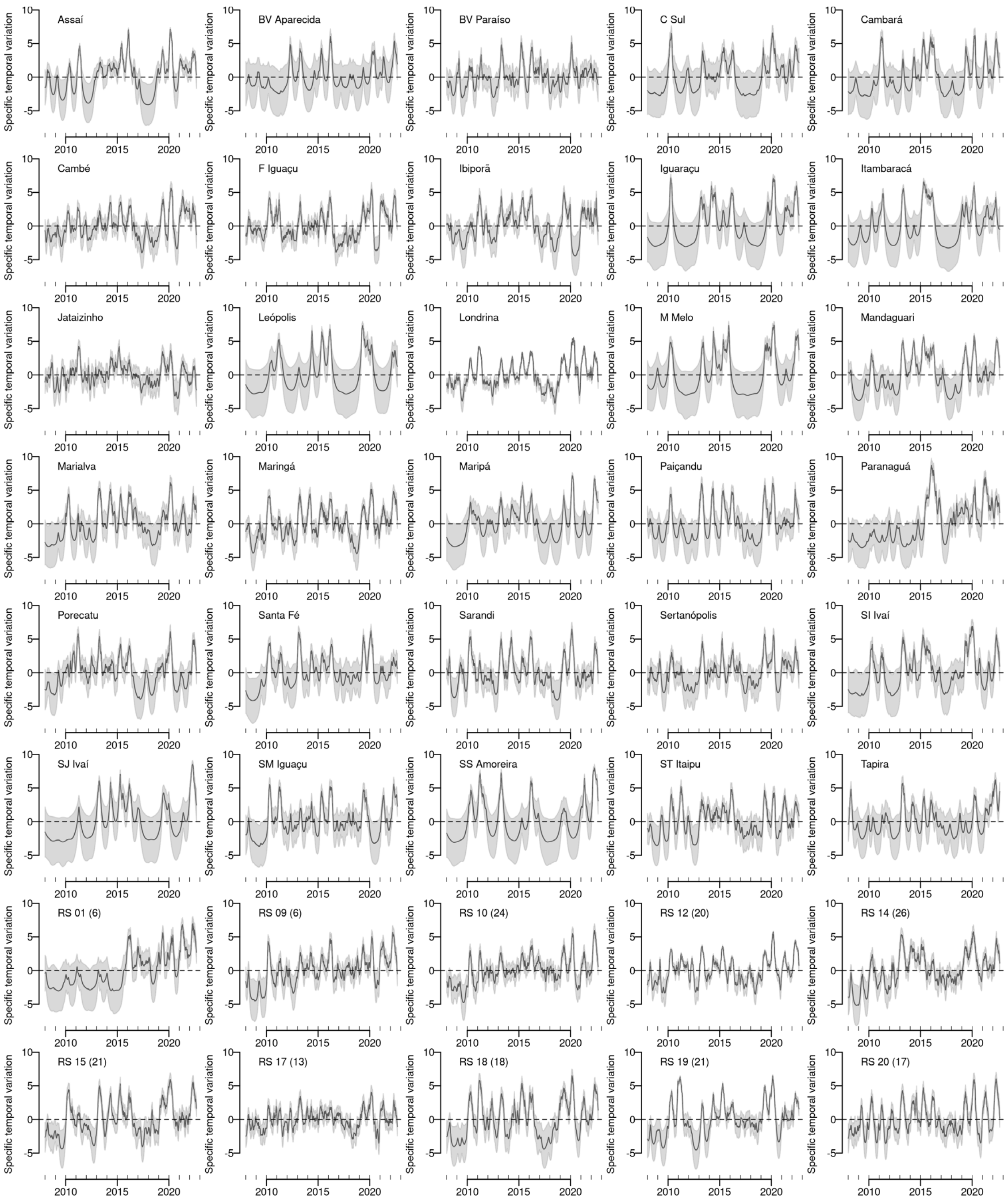

Figure 2: Time series of observed and model estimated cases of dengue, from 2008 to 2022, in 40 locations.

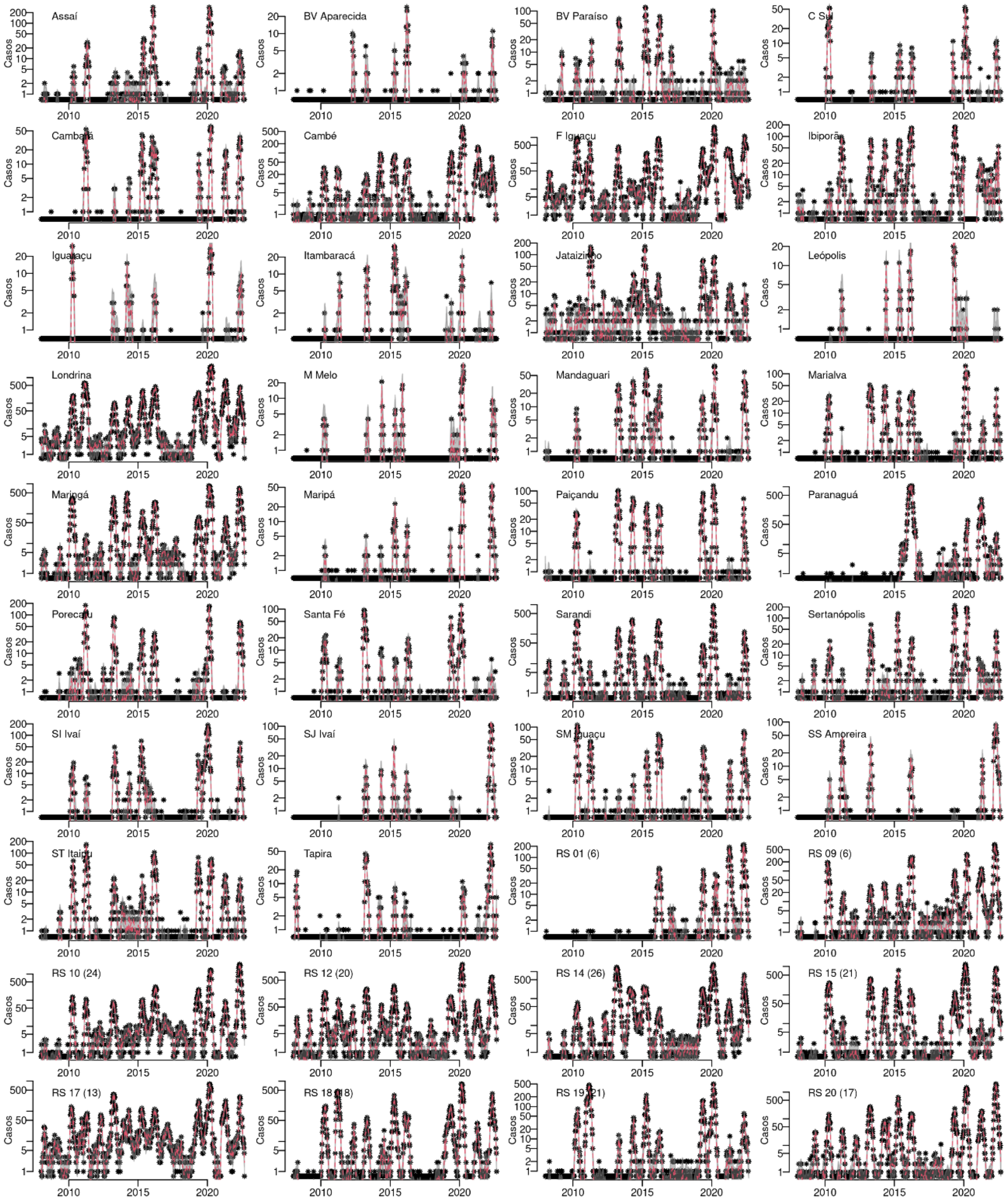

Supplement: Supplementary file 1 [file tropicalmed-11-00011-s001.zip › Supplementary Material 3.pdf]
