# Supplementary material for: Dengue Incidence Following Mass Vaccination: An Interrupted Time Series Study in Paraná, Brazil
Source: Trop Med Infect Dis. 2025 Dec 30;11(1):11. doi: 10.3390/tropicalmed11010011 (PMC12846613; doi:10.3390/tropicalmed11010011)
Supplement: Supplementary file 1 [file tropicalmed-11-00011-s001.zip › Supplementary Material 4.pdf]

Supplementary Material 4 - Time series with results of prediction scenarios

Figure 1: Weekly time series of dengue cases versus model-based estimates for vaccinated municipalities and predicted time series under two hypothetical scenarios (0% and 90% coverage), from January, 2 2019 to September, 5 2022.

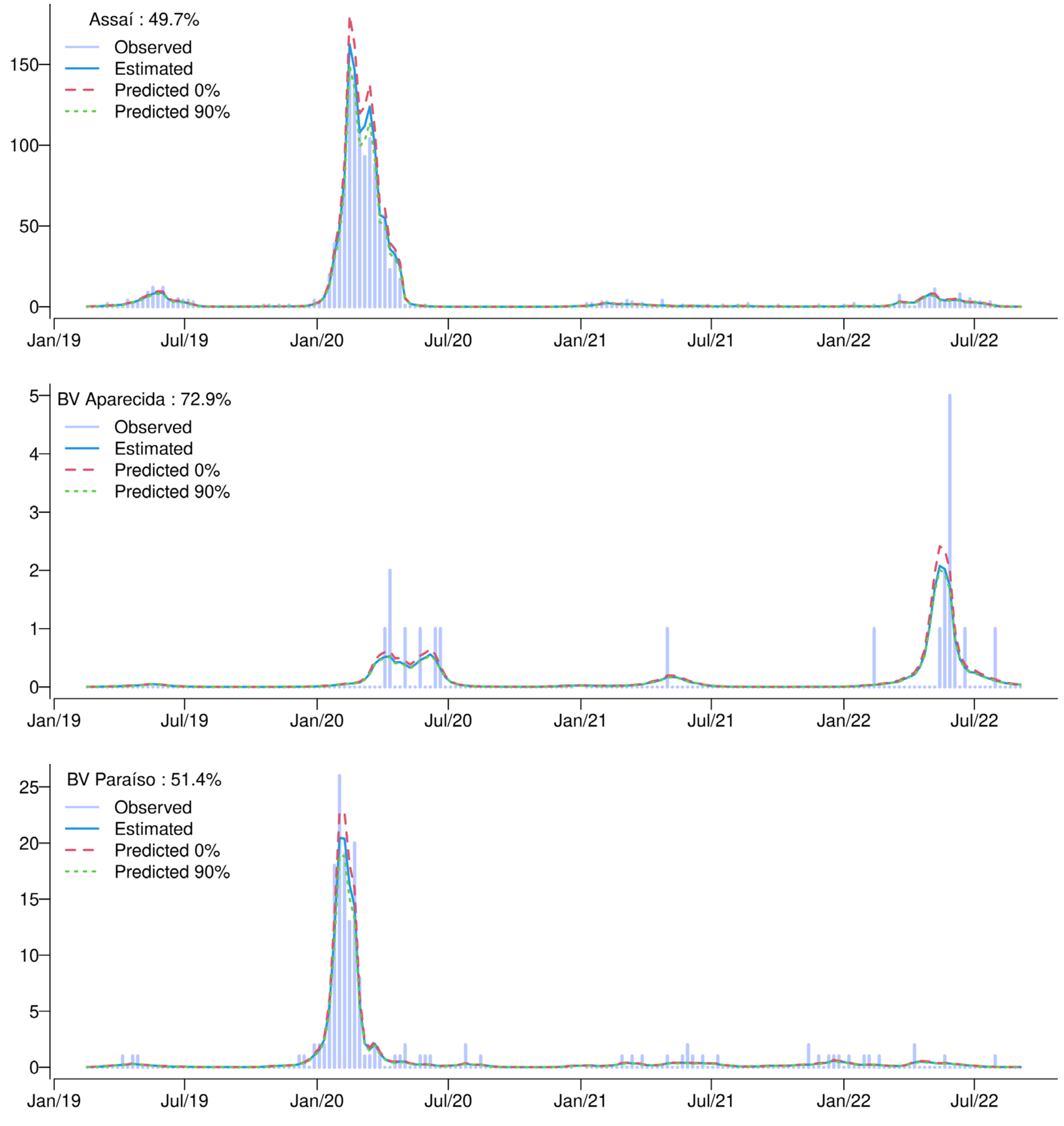

Legend: Municipality vaccine coverage rate (top left); Observed cases (light blue bars); Estimated cases (light blue solid line); Predicted number of cases under 0% coverage scenario (red dashed line); Predicted number of cases under 90% coverage scenario (green dotted line).

Figure 1: Weekly time series of dengue cases versus model-based estimates for vaccinated municipalities and predicted time series under two hypothetical scenarios (0% and 90% coverage), from January, 2 2019 to September, 5 2022. (Continued)

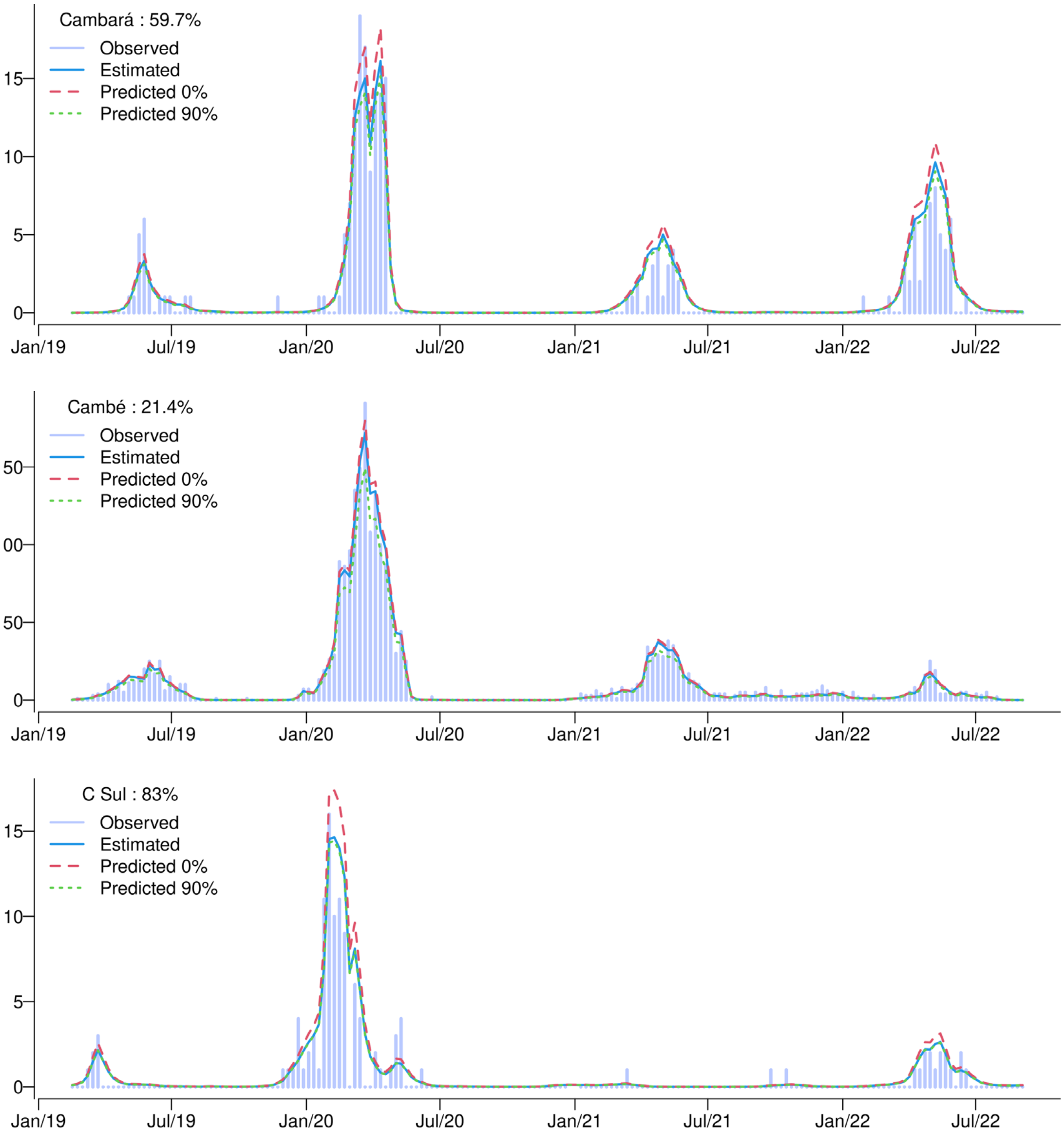

Legend: Municipality vaccine coverage rate (top left); Observed cases (light blue bars); Estimated cases (light blue solid line); Predicted number of cases under 0% coverage scenario (red dashed line); Predicted number of cases under 90% coverage scenario (green dotted line).

Figure 1: Weekly time series of dengue cases versus model-based estimates for vaccinated municipalities and predicted time series under two hypothetical scenarios (0% and 90% coverage), from January, 2 2019 to September, 5 2022. (Continued)

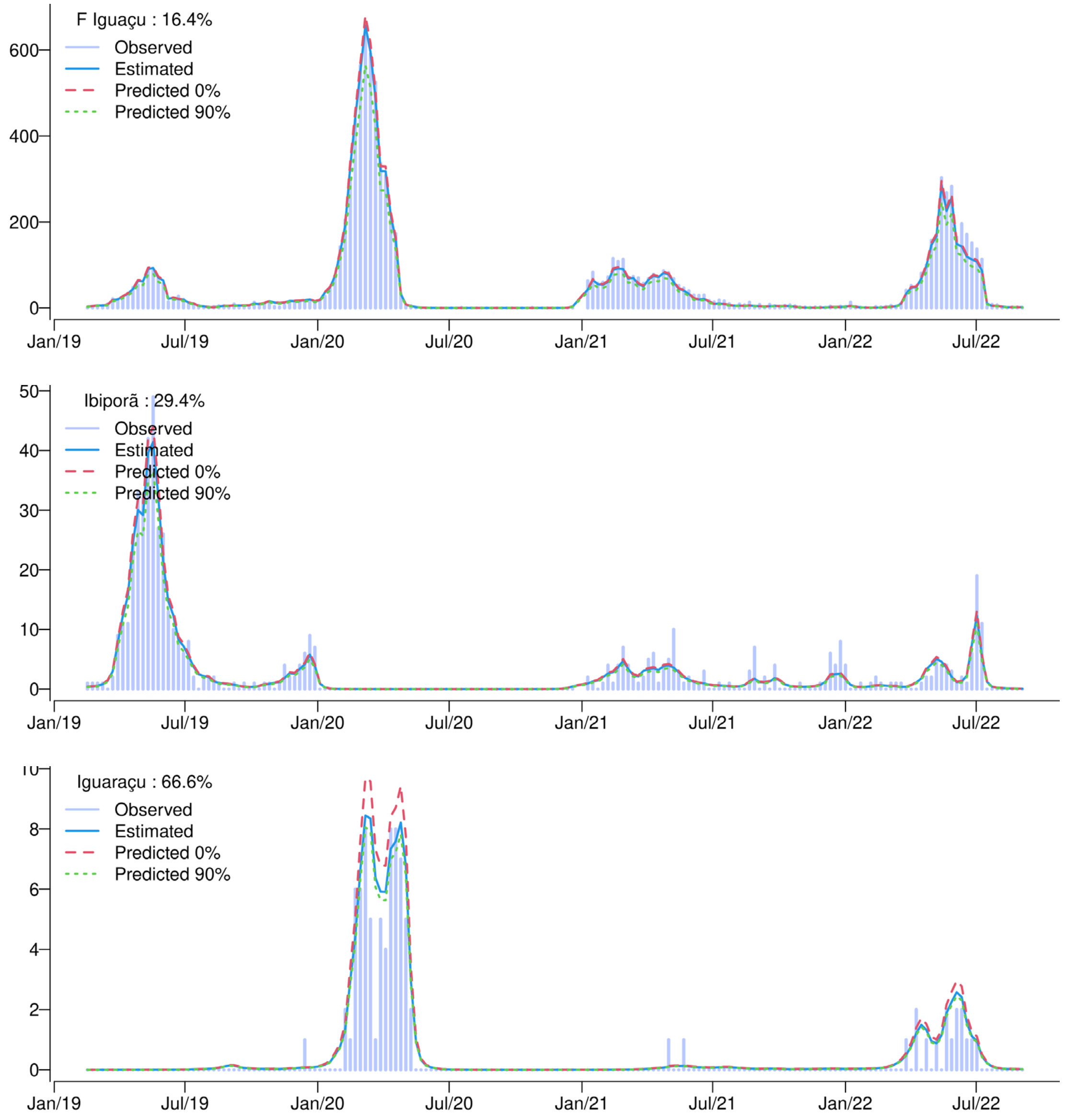

Legend: Municipality vaccine coverage rate (top left); Observed cases (light blue bars); Estimated cases (light blue solid line); Predicted number of cases under 0% coverage scenario (red dashed line); Predicted number of cases under 90% coverage scenario (green dotted line).

Figure 1: Weekly time series of dengue cases versus model-based estimates for vaccinated municipalities and predicted time series under two hypothetical scenarios (0% and 90% coverage), from January, 2 2019 to September, 5 2022. (Continued)

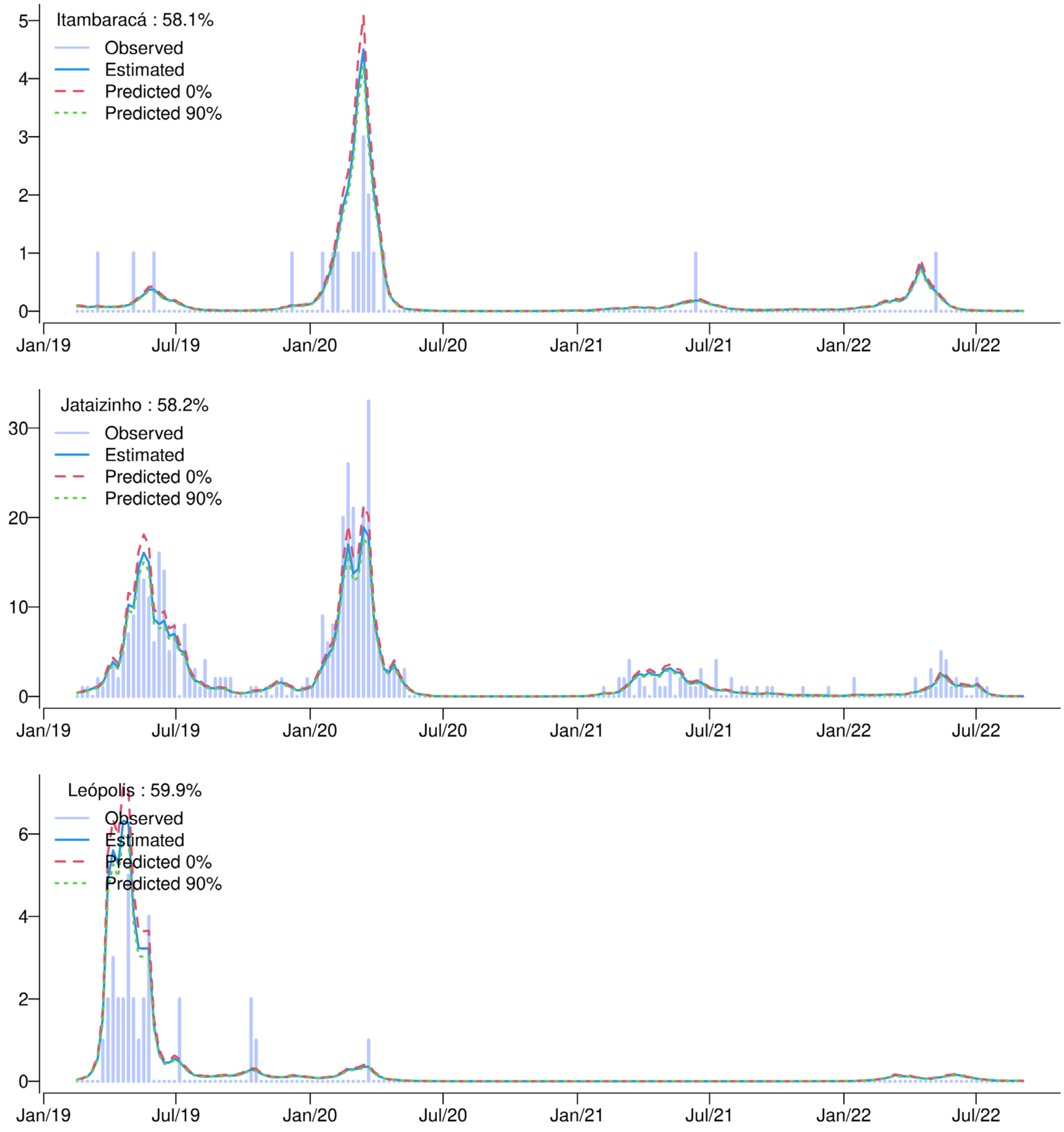

Legend: Municipality vaccine coverage rate (top left); Observed cases (light blue bars); Estimated cases (light blue solid line); Predicted number of cases under 0% coverage scenario (red dashed line); Predicted number of cases under 90% coverage scenario (green dotted line).

Figure 1: Weekly time series of dengue cases versus model-based estimates for vaccinated municipalities and predicted time series under two hypothetical scenarios (0% and 90% coverage), from January, 2 2019 to September, 5 2022. (Continued)

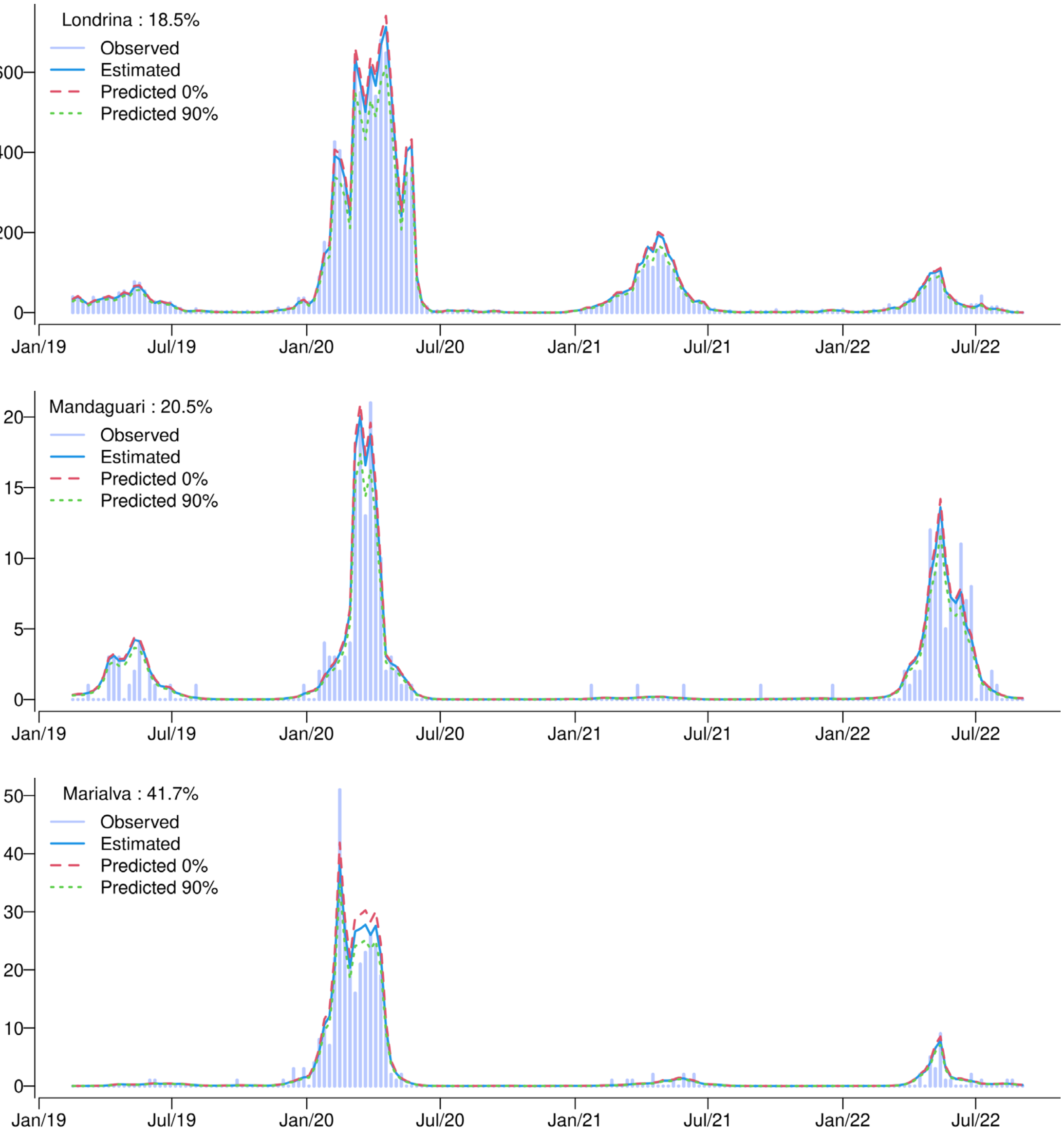

Legend: Municipality vaccine coverage rate (top left); Observed cases (light blue bars); Estimated cases (light blue solid line); Predicted number of cases under 0% coverage scenario (red dashed line); Predicted number of cases under 90% coverage scenario (green dotted line).

Figure 1: Weekly time series of dengue cases versus model-based estimates for vaccinated municipalities and predicted time series under two hypothetical scenarios (0% and 90% coverage), from January, 2 2019 to September, 5 2022. (Continued)

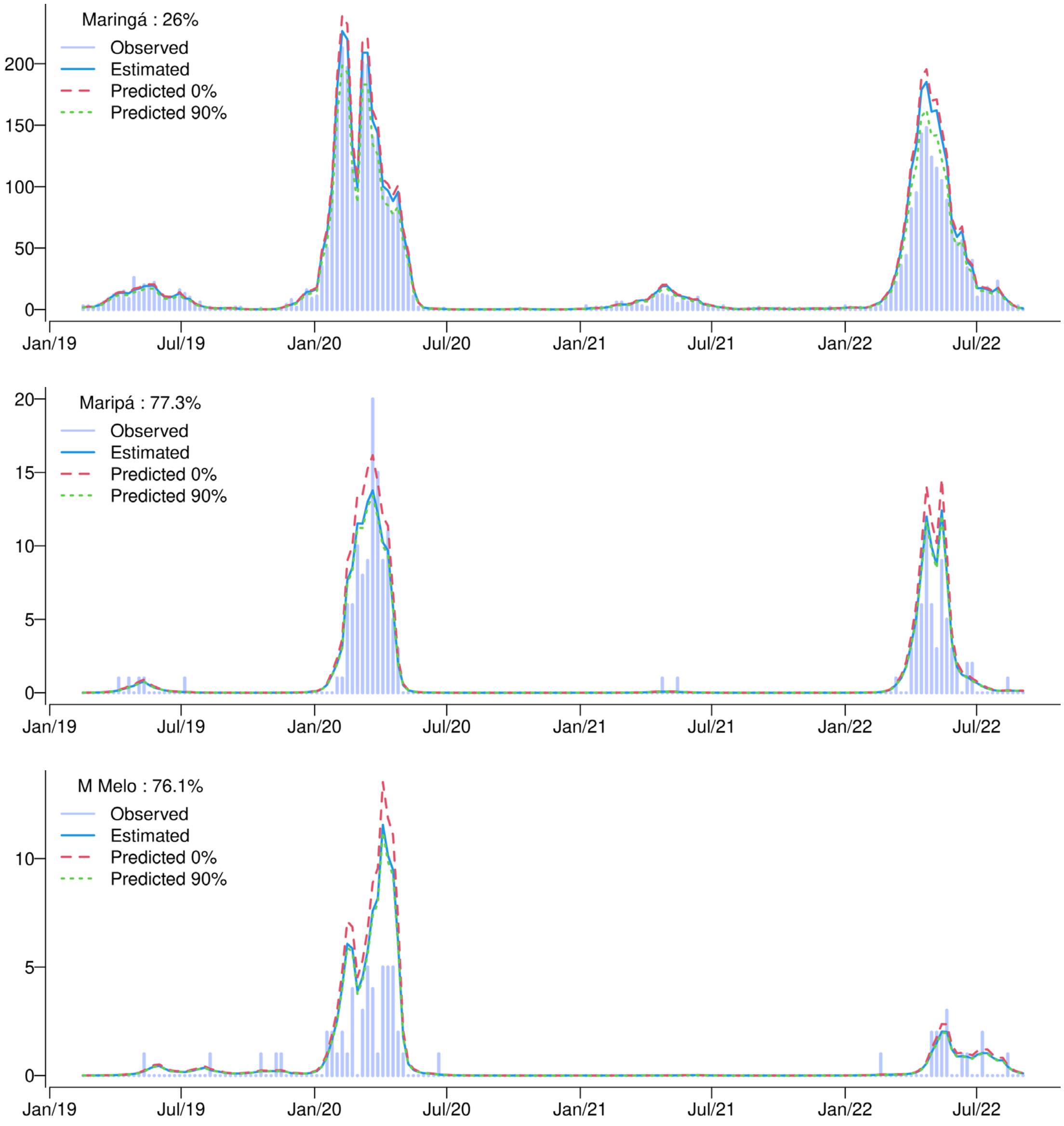

Legend: Municipality vaccine coverage rate (top left); Observed cases (light blue bars); Estimated cases (light blue solid line); Predicted number of cases under 0% coverage scenario (red dashed line); Predicted number of cases under 90% coverage scenario (green dotted line).

Figure 1: Weekly time series of dengue cases versus model-based estimates for vaccinated municipalities and predicted time series under two hypothetical scenarios (0% and 90% coverage), from January, 2 2019 to September, 5 2022. (Continued)

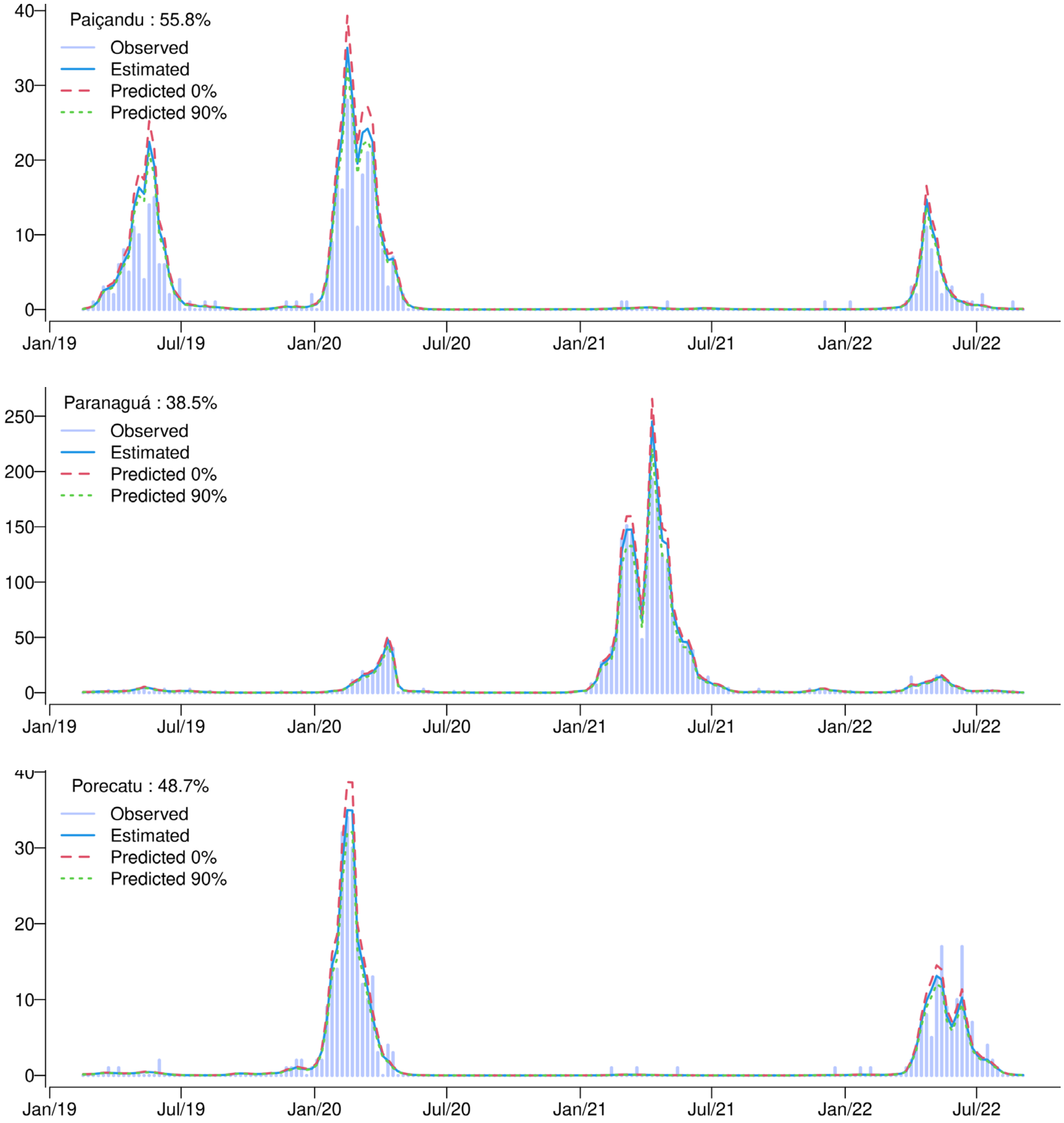

Legend: Municipality vaccine coverage rate (top left); Observed cases (light blue bars); Estimated cases (light blue solid line); Predicted number of cases under 0% coverage scenario (red dashed line); Predicted number of cases under 90% coverage scenario (green dotted line).

Figure 1: Weekly time series of dengue cases versus model-based estimates for vaccinated municipalities and predicted time series under two hypothetical scenarios (0% and 90% coverage), from January, 2 2019 to September, 5 2022. (Continued)

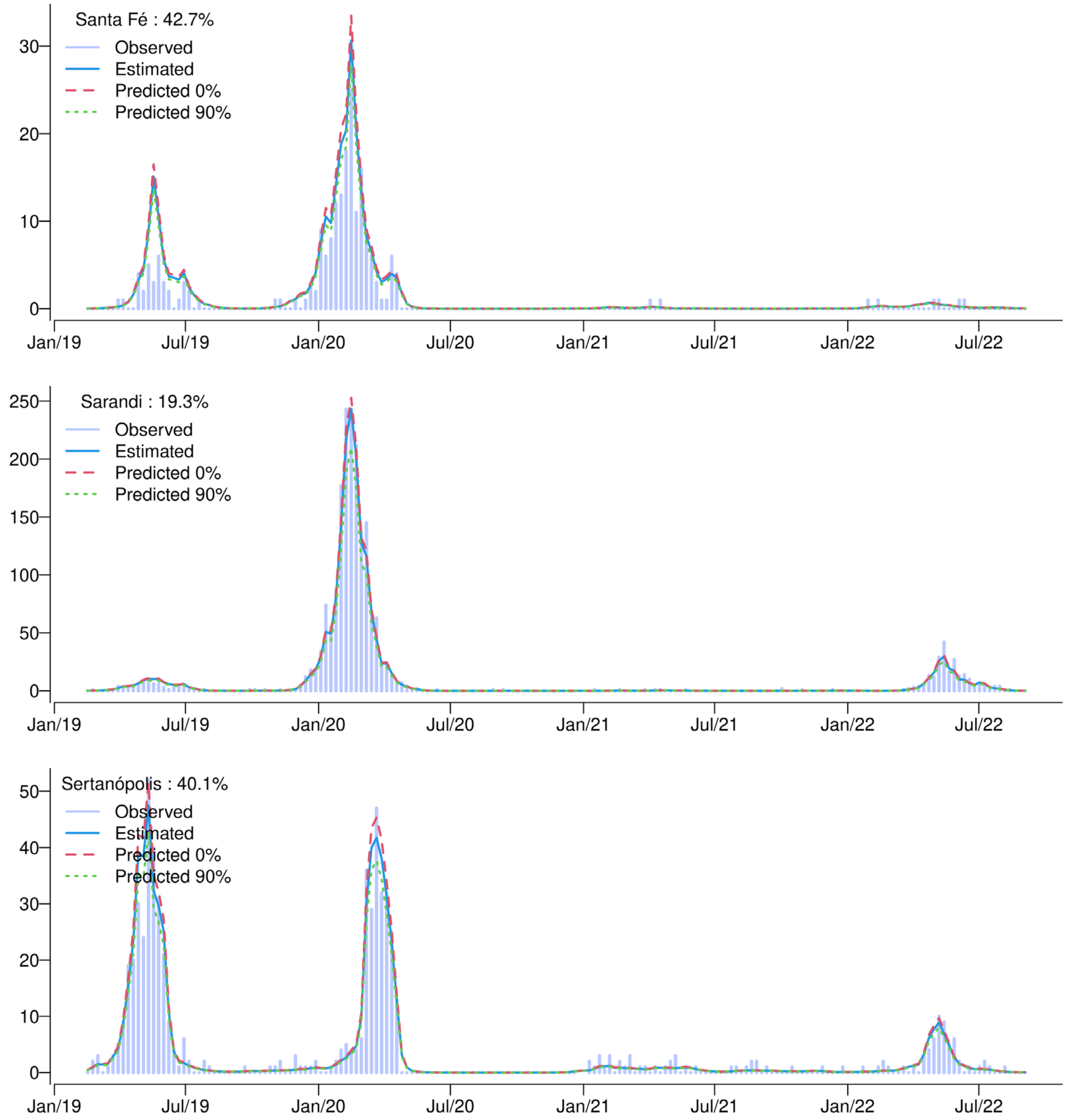

Legend: Municipality vaccine coverage rate (top left); Observed cases (light blue bars); Estimated cases (light blue solid line); Predicted number of cases under 0% coverage scenario (red dashed line); Predicted number of cases under 90% coverage scenario (green dotted line).

Figure 1: Weekly time series of dengue cases versus model-based estimates for vaccinated municipalities and predicted time series under two hypothetical scenarios (0% and 90% coverage), from January, 2 2019 to September, 5 2022. (Continued)

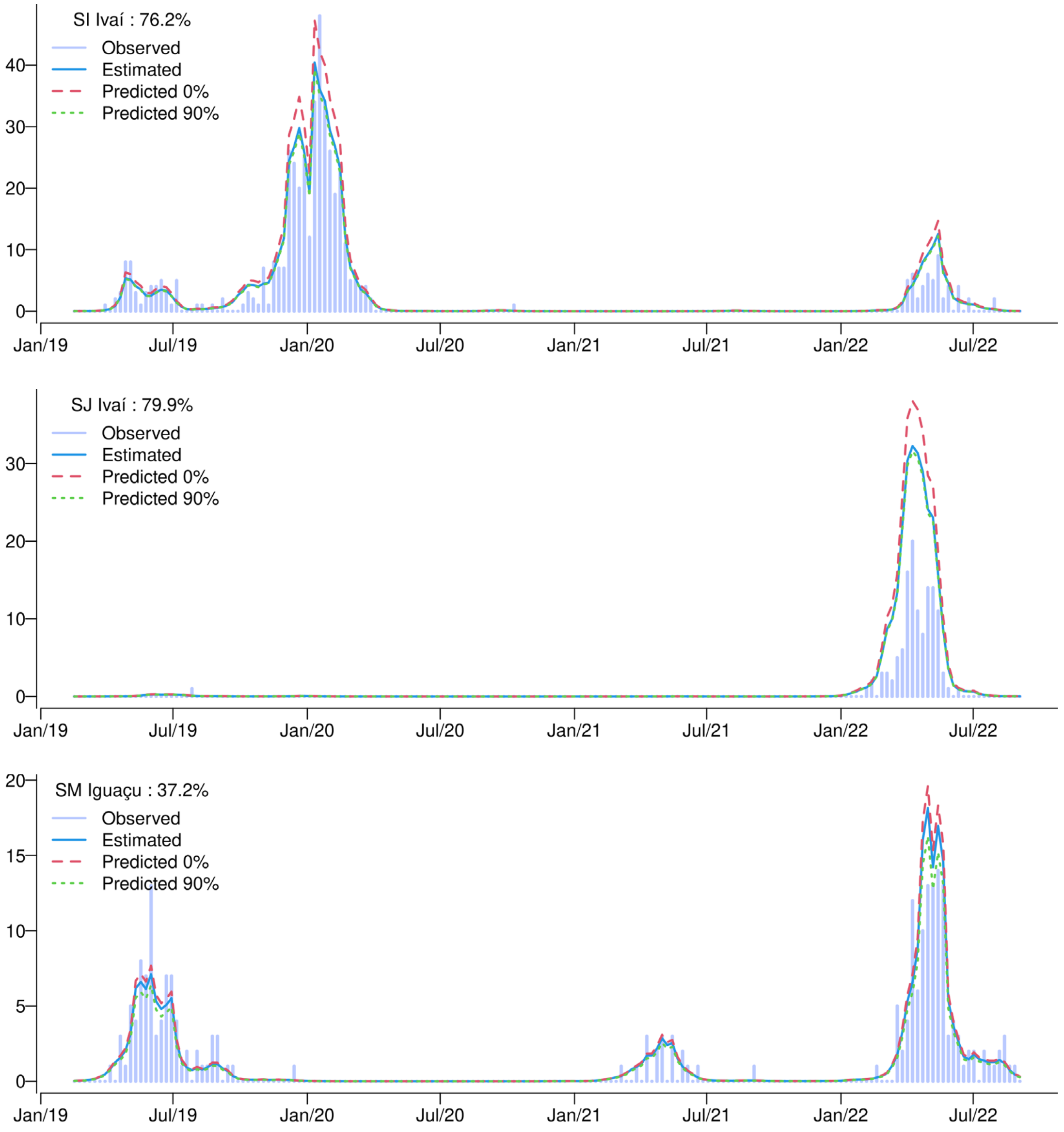

Legend: Municipality vaccine coverage rate (top left); Observed cases (light blue bars); Estimated cases (light blue solid line); Predicted number of cases under 0% coverage scenario (red dashed line); Predicted number of cases under 90% coverage scenario (green dotted line).

Figure 1: Weekly time series of dengue cases versus model-based estimates for vaccinated municipalities and predicted time series under two hypothetical scenarios (0% and 90% coverage), from January, 2 2019 to September, 5 2022. (Continued)

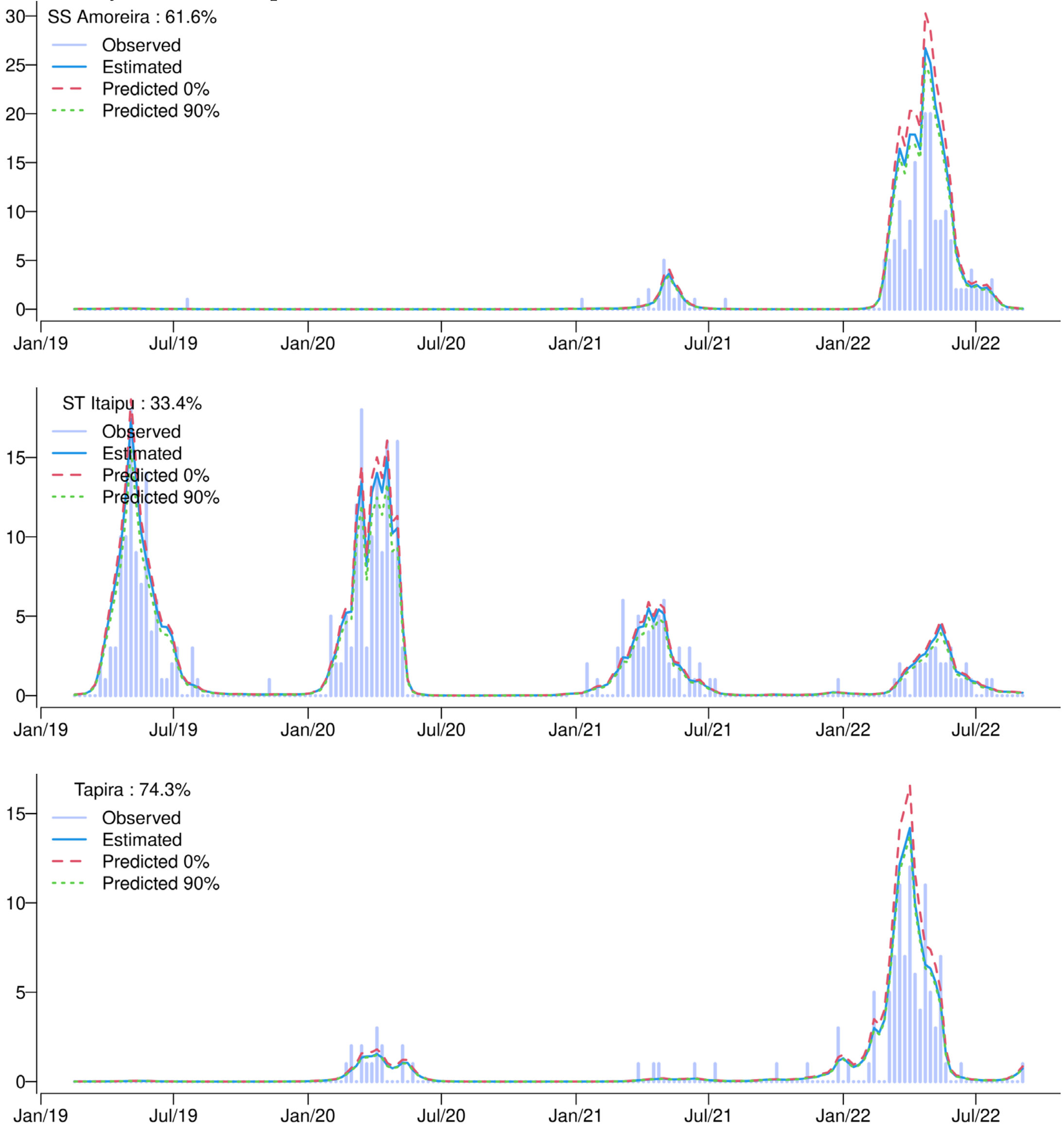

Legend: Municipality vaccine coverage rate (top left); Observed cases (light blue bars); Estimated cases (light blue solid line); Predicted number of cases under 0% coverage scenario (red dashed line); Predicted number of cases under 90% coverage scenario (green dotted line).
